# Paying for Performance to Improve the Delivery and Uptake of Family Planning in Low and Middle Income Countries: A Systematic Review

**DOI:** 10.1111/sifp.12001

**Published:** 2016-11-17

**Authors:** Claire Blacklock, Ekelechi MacPepple, Setor Kunutsor, Sophie Witter

**Affiliations:** ^1^ Claire Blacklock is Lecturer in International Public Health, Nuffield Centre for International Health and Development, University of Leeds, Leeds, UK and Honorary Clinical Researcher, Nuffield Department of Primary Care Health SciencesUniversity of OxfordOxfordUK; ^2^ Ekelechi MacPepple is Research Fellow, Department of Health Care Policy and ManagementUniversity of SurreySurreyUK; ^3^ Setor Kunutsor is Research Fellow, School of Clinical SciencesUniversity of BristolLearning & Research Building, Southmead Hospital, Southmead RoadBristolUK; ^4^ Sophie Witter is Professor, Institute for Global Health and DevelopmentQueen Margaret UniversityEdinburghEH21 6UUUK

## Abstract

Paying for performance is a strategy to meet the unmet need for family planning in low and middle income countries; however, rigorous evidence on effectiveness is lacking. Scientific databases and grey literature were searched from 1994 to May 2016. Thirteen studies were included. Payments were linked to units of targeted services, usually modified by quality indicators. Ancillary components and payment indicators differed between studies. Results were mixed for family planning outcome measures. Paying for performance was associated with improved modern family planning use in one study, and increased user and coverage rates in two more. Paying for performance with conditional cash transfers increased family planning use in another. One study found increased use in the upper wealth group only. However, eight studies reported no impact on modern family planning use or prevalence. Secondary outcomes of equity, financial risk protection, satisfaction, quality, and service organization were mixed. Available evidence is inconclusive and limited by the scarcity of studies and by variation in intervention, study design, and outcome measures. Further studies are warranted.

Accessible and high‐quality reproductive health services are critical to achieving the Sustainable Development Goals.^1^ Contraception services enable couples to limit and space births, contributing to better maternal and child health and to socioeconomic development. At the London Summit on Family Planning in July 2012 2, more than 150 global leaders made a commitment to provide an additional 120 million women and girls in the world's poorest countries with voluntary contraception services by 2020.^3^ There is, however, limited scientific evidence on the effectiveness of different finance mechanisms to increase contraception uptake and continuation.^4^

Public funding for health has traditionally taken the form of budget flows linked to indicators such as staffing levels or bed numbers, inputs (e.g. estimated drug needs), population numbers, and trends in expenditure—all subject to budget constraints. Though such budget flow systems can offer income stability, providers lack financial incentives to improve outputs or quality. Paying for performance (P4P), an alternative to the traditional budget flow system, aims to incentivize service providers by linking pay to desired provider quality and outcome indicators. P4P can target different levels of the health care structure: individual providers, health care facilities, private sector, public sector, and national or sub‐national levels.^5^ Also central to many P4P interventions are significant ancillary components, such as: education, supplies, technical support or training, monitoring and feedback, higher salaries, construction of new facilities, improvements in planning, and management and information systems.^6^

P4P is premised on the assumption that a change in provider behavior is required. If, however, barriers to services are instead associated with demand‐side factors (e.g. low affordability of services), then P4P for providers alone may not suffice, unless services become more responsive to the health needs of the population and barriers to access experienced by patients are also reduced. In addition, many theories attempt to explain the behavior of health professionals.^7^ One should also note that key factors in the utility function of the health provider (defined as wellbeing) are not limited to financial gain. Instead, additional factors include professional and social status, intrinsic factors such as altruism, cost of effort, and uncertainty about the clinical effectiveness of treatment. Other barriers to behavior change may also exist, including patient factors, technical skills, lack of resources, and organizational constraints.^7^, ^8^

P4P is an intervention with an uncertain evidence base 5, 6, 9 associated with its benefits, harms, equity, cost‐effectiveness, the role of ancillary components (e.g. increased resources), contribution of demand‐side factors,^10^ and sustainability of effects. The first Cochrane systematic review of paying providers for performance in low‐ and middle‐income countries (LMICs) in 2012 concluded that “almost all dimensions of potential impact remain under‐studied” 5, though a number of impact evaluations were underway. In this context, the aims of the current review were to identify areas in which the evidence base for financing of family planning in LMICs is strong, from which recommendations can be made, and to identify gaps in knowledge and areas for further research.

## METHODS

### Search strategy

Search terms describing the intervention (e.g., pay for performance; performance‐based funding/finance; output‐based funding/aid; results‐based funding/finance; target payment; performance‐based contracting; supply side financing/funding), outcomes (e.g., family planning; parenthood planning; contraception; fertility control; birth control), and study setting (LMICs) were used (see Appendix Table 1^1^). We searched the following electronic databases from 1994 (following the 1994 International Conference on Population and Development (ICPD), Cairo) to May 2016: CINAHL, Cochrane database of Systematic Reviews, Cochrane Central Register of Controlled Trials (CENTRAL), EMBASE, Global Health, Global Health Library‐Regional databases, IDEAS, EconLit, MEDLINE, MEDLINE In‐Process & Other Non‐Indexed Citations, Popline, WHOLIS, Social Science Citation Index (SSCI), and Science Citation Index Expanded (SCI‐EXPANDED). We searched Database of Abstracts of Reviews of Effectiveness (DARE) for related reviews. No language or publication restrictions were applied. Reference lists of retrieved articles were searched, and ISI Web of Science was searched for papers that cited included studies.

The following websites were searched: World Bank, US Agency for International Development (USAID), Management Sciences for Health (MSH), Centre for Global Development, World Health Organization (WHO), Swiss Tropical Institute, Deutsche Gesellschaft für Technische Zusammenarbeit (GTZ), KfW Entwicklungsbank, Department for International Development (DFID), Global Alliance for Vaccines and Immunization (GAVI), Global Fund to Fight AIDS, Tuberculosis and Malaria, African and Asian Development Banks, Pan American Health Organization (PAHO), CORDAID, London School of Hygiene and Tropical Medicine, Harvard School of Public Health, Ifakara Institute in Tanzania, Institute of Tropical Medicine, Belgium, RBF Health, and Health Systems 2020. International experts were contacted for relevant published or unpublished studies.

### Inclusion criteria

#### Study design

Included studies were: randomized controlled trials (RCTs); controlled clinical trials (CCTs); controlled before‐after (CBA) studies, with at least two clusters in each comparison, and appropriate choice of the control site (e.g. similar socioeconomic characteristics and/or no major differences in the baseline group); and interrupted time series (ITS) studies, with at least three measurements before and after introducing the intervention.

Impact evaluations of P4P schemes (including ancillary components) compared to any alternative (including non‐conditional financial incentives and different levels of conditional financial incentives) were included. We also included comparisons with alternatives where there may be differences in ancillary components, such as increased resources, as well as differences in P4P.

#### Population

Women and men, adolescents, married and not married, in all age groups, living in LMICs were included.

#### Participants

Participants in P4P schemes were providers of health care services in LMICs, e.g. community distributors, health workers, facilities, sub‐national organizations (health administrations, NGOs, or local governments), or national governments. Public, private, or private not‐for‐profit sectors were all eligible for inclusion.

#### Intervention

Eligible P4P interventions were: conditional cash payment to providers, conditional provision of material goods, and target payments, i.e. payments for reaching a certain level of coverage.

#### Outcome

Studies were included if a primary review outcome was reported, as shown in Appendix Table 2. For studies with missing family planning (FP) data, we corresponded with authors of the relevant studies to obtain these.

### Exclusion criteria

Studies were excluded if: they focused solely on the demand side of health care (i.e., payment to consumers such as conditional cash transfers or vouchers to beneficiaries); payments to health workers or facilities were not explicitly linked to changing patterns of performance for delivery of family planning services; or they were conducted in high‐income countries (using the World Bank classification of countries).

### Selection of included studies

Two authors independently screened titles and abstracts to identify studies that met the inclusion criteria. In the case of multiple publications, the most up‐to‐date or comprehensive study was included. Full text articles were reviewed independently by the same two authors using a standardized checklist. Disagreements and uncertainties were resolved by discussion and/or the involvement of a third author.

### Data extraction

A standardized data extraction form was used by two independent authors to extract information from the included studies. Data were entered into Microsoft Excel.

### Data analysis

For randomized and non‐randomized controlled trials and CBA studies, where possible, the difference‐in‐differences comparing intervention to control groups was calculated. In the majority of studies, we were unable to calculate risk ratios or to re‐analyze the available data. Given the limited number of studies and the heterogeneous findings across studies in relation to interventions, study designs, and outcome measures, we were unable to conduct a meta‐analysis, perform a statistical assessment of heterogeneity, or calculate averages across studies. For the same reasons, subgroup and sensitivity analyses could not be conducted. A narrative synthesis was therefore undertaken. The findings of each included study were summarized in tables, in units as reported by the investigators.

Two authors independently assigned an overall assessment of the risk of bias (high, moderate, or low) to each domain for all included studies using EPOC criteria 11 and the *Cochrane Handbook for Systematic Reviews of Interventions*.^12^ Discrepancies were discussed and final scoring agreed.

### Protocol registration

The protocol was registered with PROSPERO (2014:CRD42014014638), available at http://www.crd.york.ac.uk/PROSPERO/display_record.asp?ID=CRD42014014638.

## RESULTS

The database search retrieved 5,267 references. After screening titles and abstracts, 258 articles remained for further evaluation. Following full text review, 248 articles were excluded while 10 studies met the inclusion criteria. In addition, 3 eligible studies were identified from snowballing and grey literature. Of the 13 studies, nine were published in peer‐reviewed journals.^13^, ^14^, ^15^, ^16^, ^17^, ^18^, ^19^, ^20^, ^21^

### Characteristics of included studies

Eleven of the studies were undertaken in the Great Lakes region of Africa (Burundi, Rwanda, Tanzania, and Democratic Republic of Congo), with one in Nicaragua and one in Afghanistan (Appendix Table 3). In Rwanda, Butare and Cyangugu were the two main provinces applying P4P schemes during the period 2001 to 2004, and there was some geographic overlap between two of the studies conducted during this timeframe.^18^, ^22^ Similarly in Burundi, Bonfrer 2014 14 and Falisse 2014 16 evaluated the effects of P4P during its rollout in the period 2006 to 2010, and therefore used the same provinces for data collection. However, their sampling methods, outcome data sources, target indicators, and FP outcome measures differed.

Three studies were RCTs 15, 19, 23, nine were CBAs 13, 14, 16, 17, 18, 21, 22, 24, 25, and one was ITS.^20^ The focus of the P4P intervention was at facility or provider level, which involved a mix of public and private health facilities (health centers, hospitals, and contracted NGOs). The interventions took place in mostly rural, semi‐rural, or mixed areas. The period of data collection reported by the included studies ranged from 2001 to 2013. In only one case 23 were data collected after the intervention had ended (four months post‐intervention).

### P4P target payments

All studies used payments linked to the number of units of targeted services delivered, usually modified by quality indicators. The study in Nicaragua used a combination of P4P to providers and conditional cash transfers to patients.^25^ Targeted services mainly covered maternal health (e.g. FP uptake, prenatal and antenatal care, and institutional deliveries) and child health (e.g., childhood immunizations and growth monitoring).

The magnitude of incentive payments was not consistently reported and, where reported, varied considerably between studies and FP indicators (Appendix Table 4). In Burundi, the P4P payment for new oral and injectable FP acceptors and return clients was US$2.00, and for FP implants or intrauterine devices (IUDs) was US$5.00.^14^ Six studies conducted in Rwanda provided figures for the incentive payments for FP services, ranging from $0.18 for each one‐month contraceptive supply visit by women to US$1.83 for a new contraceptive user visit. In DRC, the price per woman who used a modern FP method was US$4.50 according to Huillery 2014.^23^ In addition to actual value, the relative value of FP unit payments compared to other incentivized services such as institutional delivery varied between studies. Where reported, the relative value of FP compared to institutional delivery was at least 40 percent in all but one study (relative value in Rusa 2009 was just 5 percent 20); in Bonfrer 2014 relative value was 100 percent 14 and in Huillery it was 90 percent.^23^

Health facilities and contracted providers were the main recipients of P4P incentives. In many cases, health facilities distributed payments among staff as salary top‐ups. Community workers also received payments from health facilities in some studies 13, 20, 22 to encourage referral of patients for child growth monitoring and institutional delivery.

### Verification of P4P target performance

The assessment and verification of performance mostly involved submission of monthly or quarterly reports by health facilities; these reports were verified by the Ministry of Health, external consultants or auditors, third party agencies, or steering committees. There were unannounced observation visits to health facilities in Burundi and Rwanda.^14^, ^17^, ^24^ In Rwanda, monitoring visits were made to the homes of randomly selected registered users of health facilities.^18^

### Setting P4P output payment values

There was no detailed information on how service performance indicators were selected or how payment values were attached to them. There were suggestions that indicator values were mostly set by the implementing authorities of the P4P schemes, which included decision makers at the Ministry of Health, provincial health authorities, and NGOs (Health Net, CORDAID) contracted to deliver the P4P schemes (Appendix Table 5).

### Funders and implementers

Sources of funding for the P4P schemes included participating governments, World Bank, EU, Inter‐American Development Bank, bilateral aid agencies (Swedish International Development Agency), international NGOs (CORDAID), and UNFPA. In Burundi, of the total cost of P4P funding, 52 percent was contributed by the Government of Burundi, 28 percent by the World Bank, and 20 percent by other donors.^14^ With the scale up of performance‐based financing (PBF) to the whole country in 2010 and its becoming national policy, the Government of Burundi (Ministry of Health) was then providing the majority of the funding for the program (1.4 percent of the national budget was allocated to PBF and other health programs annually).^14^ Implementing partners of the P4P schemes included participating governments (Ministries of Health) and NGOs (CORDAID, Health Net International).

### Ancillary components

All studies featured ancillary components in addition to the main P4P intervention. The most common ones were training, supervision, and feedback. In DRC and Rwanda, user fees were also lowered in health facilities, and facilities were given greater autonomy in management, setting of user fees, and payment allocations to staff.^18^, ^19^, ^20^, ^21^, ^22^, ^23^, ^24^ In DRC, informal taxation on the health facilities by local health authorities was eliminated, as a result of monthly payments to the local health authorities under the scheme.^21^ In the majority of cases, the P4P scheme provided additional resources. In two schemes, increased funding was also provided to the control districts to match the approximate cost of PBF (DRC and Rwanda).^17^, ^23^, ^24^ In one study, the control provinces received alternative forms of support: one received increased staffing from the government, the other received supervision by an EU‐funded project.^22^ In Tanzania, district and regional managers received significant performance‐related payments according to the performance of health facilities in their districts.^13^

### Contextual factors

In addition to ancillary components of the P4P schemes, some studies were undertaken amid external contextual circumstances that may have affected the outcomes. In May 2006 (before the start of the P4P intervention), user fees for pregnant women and children under five years were abolished nationwide in Burundi. In response, health facilities received payments from the government for services provided free of charge to replace the lost income. In Rwanda, the P4P intervention operated alongside a *mutuelles* community health insurance scheme (implemented from 2002), which covered the majority of the population studied, and a national sensitization campaign by the media and civil authorities led to strengthening of other components of the health system, such as data collection, monitoring, and integrated supervision.^20^ Hence significant contextual differences were present within and between studies. These included community factors (baseline prevalence and uptake of services), financial factors (such as free health care initiatives, community health insurance schemes, and reduction of user fees), and national system factors (e.g., researchers noted the supportive national administration in Rwanda 18).

### Completeness of outcome data collection in studies

We have focused on FP uptake, the primary outcome of this review, and on secondary outcomes described above. A number of the studies reported selected output indicators based on the availability or completeness of the data. In Burundi, for instance, of the 23 output indicators that were set, Bonfrer 2014 could assess only a subset of six.^14^ Similarly in Falisse 2014, of the 42 impact indicators, only six were assessed (Appendix Table 4).^16^

### Quality of studies, including design and implementation

#### Robustness of findings

The risk of bias for each study is summarized in Appendix Table 5. Three studies were assessed as having high risk of bias in six or more domains;(16, 18, 22) two studies had high risk of bias in five domains;(14, 21) two had high risk of bias in four domains;(13, 25) two had high risk of bias in three domains;(17, 24) and three (RCTs) had high risk of bias in one or two domains.^15^, ^19^, ^23^

Several significant limitations were demonstrated in studies: selective reporting of outcomes because of unavailability of data, confounding programs such as the elimination of user fees and ongoing community health insurance schemes, heterogeneity in the design and implementation of P4P intervention at different clusters, and authors of studies being involved in design of interventions (Appendix Table 5). Of particular note is the heterogeneity in the design and implementation of interventions and evaluations. Examples of implementation challenges reported by authors included the finding that the majority of health workers in intervention facilities in Afghanistan did not recognize that they had received performance payments during the intervention period.^15^ Implementation of the evaluation process also faced difficulties, such as reallocation of facilities following recentralization,^17^, ^24^ poor‐quality HMIS data, data collection occurring in different seasons,^22^ and non‐comparable conditions among control clusters.^22^

### Primary review outcomes

#### Use of FP services and FP prevalence

In Burundi as reported by Bonfrer 2014, pooled data showed an absolute increase of 2 percentage points for the intervention provinces compared to control provinces in the use of modern FP services in the period 2006–2008, before and after the introduction of P4P.^14^ In contrast, Falisse 2014 found that P4P was not significantly associated with the use of FP (IUDs).^16^ In DRC, Huillery 2014 found no impact of P4P on the use of modern FP: only 5 percent of women aged 15–49 used a contraceptive method.^23^ Soeters 2011 also found no significant increase in the household use of FP comparing the intervention to the control group (12 percent increase versus 8 percent increase).^21^ In Nicaragua, a combination of demand and supply payments for performance was associated with an increase in use of FP methods by 5 percentage points from an initial level of 24 percent among women aged 12–49 years, with a net impact three times greater among women aged 30–40.^25^ In Rwanda, no statistically significant overall difference was found in the use of modern contraceptives when comparing the intervention groups to controls.^19^, ^24^ No significant differences were found in the use of family planning in Afghanistan or Tanzania.^13^, ^15^

#### New FP users

In Rwanda, Meessen 2006 reported increases in new subscribers to FP in each P4P intervention district compared to the controls.^18^ However, except for one intervention district (Cyangugu), the FP coverage rates in the intervention districts declined compared to rates in controls. Soeters 2005 also found an increase in new acceptor FP coverage rates of 2.8 percentage points in the intervention provinces compared to 0.2 percentage points in the control provinces, though with very low absolute coverage (3.9 percent).^22^

None of the studies reported on changes to continuation, switching, unmet need, or change in method mix.

### Secondary review outcomes

#### Cost, efficiency, cost‐effectiveness

Eight studies provided information on the total cost of the P4P intervention (Appendix Table 6). The range of investments per capita per year was US$0.20 20 to US$2.00.^21^, ^22^ In the study by Meessen 2006 conducted in Rwanda, the annual cost was around US$93,000 for three years.^18^ In DRC as reported by Regalia 2007, the total project cost (for both demand and supply sides) was US$22 million over three years.^25^

Payments varied considerably in magnitude at facility level, although the size of studies varied and the specific costs for FP are unknown. In absolute terms, health centers in Falisse 2014 received €1,100–2,000 per month,^16^ while in Huillery 2014, also in Burundi, the average was $550 per facility per month.^23^ In Soeters 2011, the payments varied from $200 to $4,000 per facility per month, leading to a 36 percent increase in facility revenues (compared to a 14 percent increase in the control areas).^21^ In Gertler 2012 the increase in funding at the facility level averaged 25 percent.^24^ In Burundi, payments averaged 40 percent of facility revenues.^14^ In Tanzania, health centers received maximum payments of between US$820 and US$6,700, depending on type of facility. In addition, district and regional managers could receive up to US$3,000 per payment cycle.^13^

In these studies, the scale of payments was significant at the facility level. However, it appears that the bulk of payments were passed on to staff, rather than being reinvested in facility costs. In Soeters 2005, 5 percent of P4P funds went to facilities in one district, compared to 60 percent in the other.^22^ In Priedeman Skiles 2013, the proportion was 25 percent,^19^ while in Rusa 2009 the funds going to facilities grew from 8 percent in the first year to 38 percent in the third.^20^ This increase may reflect the context of underfunded staffing in the region. P4P in most cases led to significant additional resources for staff, whose remuneration rose from $75 in 2006 in the P4P facilities to $262 in 2011.^16^ In Huillery, P4P was associated with a 34 percent reduction in staff pay,^23^ whereas Regalia 2007 reports that pay to staff in contracted facilities was 30–50 percent higher than for comparable Ministry staff.^25^ In Gertler 2012, 77 percent of revenues went to staff, leading to an 83 percent increase in pay.^24^ In Meessen 2006, 39 percent of staff income was derived from P4P,^18^ while in Rusa 2009 the proportion varied from 39 percent to 84 percent.^20^ In Priedeman Skiles 2013, staff received a 38 percent salary top‐up,^19^ while Soeters 2005 reported a 40 percent contribution in one district from P4P.^22^ At least 75 percent of the payment was passed on to health workers in Binyauka 2015.^13^ In Afghanistan, 10 percent of payments were retained by NGO offices, with the remainder distributed to health workers, leading to a 6–28 percent increase above base salary.^15^

No study reported on cost‐effectiveness.

###  

#### Client satisfaction with services

Two studies conducted in DRC, one study in Burundi, one in Tanzania, and one in Afghanistan reported on patient satisfaction. Huillery 2014 reported no impact of P4P on patient satisfaction or the use of health facilities.^23^ Bonfrer 2014 also cited no significant change in patient‐reported quality.^14^ In contrast, Soeters 2011 observed a statistically significant improvement in patient‐perceived quality composite scores in intervention groups compared to control groups (8 percent increase versus 17 percent decrease).^21^ Binyauka 2015 found no effect on patient satisfaction for targeted services. Alarmingly, despite a significant improvement in satisfaction for non‐targeted services, a significant reduction in outpatient attendance was seen at dispensaries, the most common type of facility.^13^ Engineer 2016 found no effect of P4P on client satisfaction.^15^

#### Quality and range of care and services

Five studies reported an assessment of quality of care and services. The results were mixed. Pooled results showed an increase of 17 percentage points in the overall facility quality score in Burundi;(14) significant improvements in three measures of service quality: time with patients, more complete history and examination, and counselling patients in Afghanistan;(15) no effect of P4P on technical quality and a negative impact on quality of equipment and infrastructure in DRC;(23) and in Rwanda, the intervention group outperformed the control group in terms of a composite quality score (75 percent vs 47 percent).^22^ Rusa 2009, however, reported an improvement in quality of services in both intervention and control groups.^20^

#### Impacts on service organization

P4P seeks to change incentives and resources, which may have effects on the organization of services. In addition, administration of P4P requires regular institutional data collection, monitoring, and reporting. Except for Bonfrer 2014 and Falisse 2014, all studies reported on changes to organizational services. New health facility autonomy in spending, or setting of user fees, was reported in three studies: two in DRC and one in Rwanda.^19^, ^22^, ^23^ A new practice of sub‐contracting services was reported in studies from DRC, Nicaragua, and Rwanda.^19^, ^22^, ^25^ Intervention facilities in some studies became more focused on community outreach 13, 23, 25 and made greater efforts to provide preventative health sessions.^23^ Changes in professional structures and behavior were reported in studies from Rwanda, such as novel management structures, revision of job descriptions, and greater focus on supervision and accountability.^18^, ^20^, ^22^, ^25^

#### Unintended effects

In DRC, Huillery 2014 reported that increased efforts on targeted services did not come at the expense of non‐targeted services.^23^ However, the P4P intervention in Tanzania was associated with a significant reduction in non‐targeted outpatient visits at dispensaries, which represent the majority of health facilities in the region.^13^ Significantly lower salaries were reported in the P4P group in DRC as health facilities reduced user fees to attract patients, leading to reduced income.^23^ In Tanzania, the P4P intervention led to staff spending 17 percent of their time each month on data generation and verification.^13^

#### Equity

The effects of P4P on equity were reported in five studies. 13, 14, 15, 17, 19 In Burundi, there was no strong evidence of any differential impact across socioeconomic groups. Priedeman‐Skiles also found no difference in the effect of P4P by wealth level in Rwanda.^19^ However, an analysis by wealth group by Lannes found that an overall non‐significant impact of P4P on family planning was masking marked inequity in effect. Indeed, contraceptive use increased significantly in the upper wealth group with P4P, but no significant change occurred in the lower wealth group (possibly confounded by a preceding strategy to increase access to family planning services for the poorest).^17^ The findings of Regalia 2007 implied that a combination of P4P and CCT targeted at the poorest households improved access to health care.^25^ The impact on other socioeconomic groups was not explored. Falisse found no evidence of P4P equalizing differences between urban and rural health centers.^16^ There was no effect of P4P on equity of care use in Afghanistan.^15^ There was no reporting on meeting the needs of more marginalized sub‐groups, such as adolescents.

#### Financial risk protection

Only one study looked specifically at financial risk protection. In DRC, though there was more spending by patients in the intervention than control areas, quality was perceived to be better, and there was no overall catastrophic health spending^2^ post‐intervention in the P4P group.^21^ User fees for targeted services were significantly reduced.^23^

#### Provider satisfaction and behavior change

Effects of P4P on provider satisfaction were specifically reported in four studies.^15^, ^21^, ^22^, ^23^ In Rwanda, staff expressed greater satisfaction in the intervention provinces. In DRC, staff expressed decreased job satisfaction in the P4P intervention group and attendance was lower compared to the control group when P4P was concluded.^23^ Managers were reported to be more satisfied with health authority supervision in the intervention than in control districts.^21^ In Afghanistan, there was no effect of P4P on provider motivation or job satisfaction.^15^ In two cases, P4P was found to attract better‐qualified staff to intervention facilities 16, 21 and staff were reported to be present more often.^23^

#### Sustainability and scale up

In two cases, sustainability issues were explicitly mentioned by the authors.^16^, ^22^ Falisse 2014 commented on the unknown sustainability of the incentive mechanism itself and the financing of the scheme.^16^ Soeters 2005 reflected that once performance improved, funding must also increase to keep up with increased volume of service provision.^22^

In Burundi and Rwanda, P4P was scaled up nationally.^16^, ^20^ In the study by Huillery 2014 conducted in DRC, the P4P program was terminated in September 2012.^23^ In Nicaragua, the P4P program was not continued beyond the external donor funded time scale.^25^

#### Fertility changes

Bonfrer 2014 reported a reduction in the proportion of households with children born in the past 12 months; however, this was a pooled before–after result for both intervention groups between 2008 and 2010, instead of a difference‐in‐differences between Phase I and Phase II sites between 2006 and 2008, when Phase II sites had yet to implement the scheme.^14^

#### Health outcomes

The impact of FP uptake on health outcomes (e.g. sexually transmitted infections, maternal deaths averted) was not reported or estimated.

### Excluded studies

The excluded studies are listed in Appendix Table 7. Reasons for exclusion were inadequate study designs 26, 27, 28, 29, 30, 31, 32 and studies that met criteria but had too few data points.^33^, ^34^, ^35^, ^36^

## DISCUSSION

FP outcome measures (use of FP services, new FP users, and FP prevalence) showed mixed results following a P4P intervention. P4P was associated with an increase in the use of modern FP in two studies 14, 17 and an increase in subscriber and coverage rates for FP in two others.^18^, ^22^ However, eight studies reported no impact of P4P on use of modern FP or on FP prevalence rates. A combination of P4P and conditional cash transfers showed an increase in use of FP methods in Nicaragua.^25^ Findings were mixed for reported secondary outcomes of equity, financial risk protection, satisfaction with services among clients, quality of care and services, provider satisfaction, and impact on service organization. There were gains for some outcome measures but no improvement for others.

FP was targeted alongside other maternal and child health services in the identified studies. Because the FP element was part of a wider incentivized service package, limited details about FP service organization, usual cost and availability of pharmaceuticals, method mix, or community contexts and how these various factors were addressed by the intervention were reported. It is possible that a more focused P4P approach targeting FP alone might have shown different results, with unknown implications for overall health service delivery. Mixed results may also be attributable to calibration of payments or may reflect demand‐side factors that impede uptake, as suggested by Bonfrer 2014.^14^ A number of authors highlighted the need to consider the barriers to use of health services that are not addressed by P4P,^23^ as well as the interaction between P4P and related policies, such as changes in user fees.^16^ Regalia 2007 was the only study in which demand‐side barriers were specifically addressed through conditional cash transfers.^25^ However, in Rwanda, complementary measures such as reduction in user fees, community insurance schemes, and subcontracting of community health workers to refer target patients to facilities are described.^20^

In some studies that showed minimal, mixed, or no effects on FP outcomes, there was nevertheless an increase in institutional deliveries in P4P intervention sites. This was seen in studies from Rwanda,^17^, ^18^, ^19^, ^20^, ^22^, ^24^ Tanzania,^13^ and Burundi.^14^ A variety of factors may have contributed to observed differences, including changes to user fee requirements, targeted community outreach, and additional unknown patient, provider, and community factors. Targeted payments for institutional delivery in these studies were usually higher than for FP. It is also possible that overall FP uptake may have been unequally distributed, such as in Lannes, which showed that an overall non‐significant change in modern contraceptive use after P4P in Rwanda masked a large increase in the wealthiest group and a negative effect in the poorest group.^17^

While the theory of change resulting from P4P focuses on incentivizing and enabling providers, the knock‐on effects on user costs may be an important mechanism. According to Huillery 2014, for example, P4P led to increased effort to attract patients (including lowering user fees), although this did not lead to increased use.^23^ In all contexts, it would be useful to understand baseline charges for and provision of FP services––whether these are provided free of charge to users through public outlets, whether they are included in health insurance packages, and whether they are part of integrated health service provision. Studies did not provide this kind of contextual information.

The institutional and national policy context in which these programs were being rolled out is also important to consider. For example, it is possible that the high‐profile support given to FP by Rwanda's President boosted use generally, masking intervention impact in Gertler 2012.^24^, ^37^ As Priedeman Skiles 2013 reports, modern contraceptive use among married women in Rwanda increased from 10 percent in 2005 to 45 percent in 2010 19 and the equity gap between poorest and richest wealth quintiles declined from 21 percent in 2005 to 7 percent in 2007. No consistent pattern of higher service use was evident, however, in P4P versus control populations for modern contraceptive use. It is also worth noting that half of the studies took place in Rwanda, which is widely recognized for its capability to implement programs well.^38^

The issue of coercion needs more attention in the literature. P4P, if it provides higher monetary incentives for specific methods, may restrict FP choice. It is recommended that clients are offered, either directly or through referral, a broad range of methods and services to meet their individual needs and preferences. While the literature on conditional cash transfers has acknowledged this risk, there is no indication that P4P designers and implementers are aware of and mitigating this risk.

This review is the first to systematically examine the effects on family planning uptake and outcomes of P4P programs aimed at providers. It complements and updates other recent related reviews, such as: a 2012 Cochrane review 5 with a broader services focus; a review of performance‐based incentives in community‐based FP programs, which mainly identified studies of sales commissions to community agents;^38^ a review of health services for mothers and newborns;^39^ a review of quality of maternal and child health care;^40^ and a review of financial incentives for uptake of FP among users.^41^ It is broadly consistent with other systematic reviews of P4P, which highlight the need for larger numbers of robust studies and for tracking a broader range of indicators to capture some of the system effects and possible unintended consequences.^42^

The timing of the studies is notable. Despite the wider timeframe of the search (from 1994), all studies date from the past 15 years, reflecting the fact that this is a growing field, with more impact evaluations expected to be published over the next few years.

### Limitations

Only a small number of studies were eligible, mostly focused on Central Africa. The generalizability of findings is therefore limited. Study contexts were predominantly post‐conflict and with very low baseline coverage levels for FP. Many studies had primary outcomes of institutional deliveries rather than FP use. The studies were therefore likely to be powered and designed accordingly, which may have affected the significance of FP results. In addition, while the review protocol envisaged a range of P4P designs, all of the eligible studies presented performance‐based financing (PBF) models, using payments at facility level to reward quantity of services provided, commonly with a quality adjustment measure. We are therefore unable to comment on other approaches, such as P4P programs targeted at national or health worker levels. Limited evidence on cost‐effectiveness remains a problem, as does the absence of a clear link with health outcome measures related to FP. A recent review focusing on economic evaluations of P4P found that evidence on the efficiency of P4P is scarce and inconclusive and that P4P efficiency could not be demonstrated.^43^ All studies had a high risk of bias in at least one domain of the risk of bias tool, with six studies having high risk of bias in four or more domains. None of the included studies reported sample size power calculations. In the majority of studies, we were unable to calculate risk ratios or re‐analyze the data. The limited number of studies and heterogeneity also meant that meta‐analysis and sub‐group analysis were not possible.

## CONCLUSIONS

On the basis of the available evidence, the impact of P4P on FP uptake and associated health outcomes in low‐ and middle‐income settings is inconclusive. The interpretation of study results is limited by variation across studies in relation to intervention design, study design and outcome measures, and the limited number of eligible studies.

More robust and comprehensive studies are warranted, including more exploratory methods such as realistic evaluation 44 that can help to unpack mechanisms of change, or explain reasons for lack of change.

## Supporting information

Disclaimer: Supplementary materials have been peer‐reviewed but not copyedited.

Appendix Table 1: Search strategy (Medline)

Appendix Table 2: Primary and secondary outcomes of review

Appendix Table 3: Characteristics of included studies: the intervention

Appendix Table 4: P4P indicators, evaluation data and main outcomes

Appendix Table 5: Robustness of included studies

Appendix Table 6: Secondary outcomes

Appendix Table 7: Excluded studies

## Footnotes

1 Appendix tables are available at the supporting information tab at wileyonlinelibrary.com/journal/sfp.

2 Defined in the cited study as a household spending more than 30 percent of household assets on a disease episode.
