# Supplementary material for: Paying for Performance to Improve the Delivery and Uptake of Family Planning in Low and Middle Income Countries: A Systematic Review
Source: Stud Fam Plann. 2016 Nov 17;47(4):309–24. doi: 10.1111/sifp.12001 (PMC5434945; doi:10.1111/sifp.12001)
Supplement: Supplementary file 1 — Appendix Table 1: Search strategy (Medline) [file SIFP-47-309-s001.docx]

**Appendix Table 1: Search strategy (Medline)**

| **Searches** | **Results** |
| --- | --- |
| exp maternal health services/ or maternal welfare/ or maternal mortality/ | 46273 |
| Maternal-Child Health Centers/ | 2166 |
| perinatal care/ or postnatal care/ or preconception care/ or prenatal care/ | 27055 |
| pregnancy/ or pregnant women/ or mothers/ | 726268 |
| child welfare/ or infant welfare/ or child mortality/ or infant mortality/ or perinatal mortality/ or exp child health services/ | 64196 |
| (preventive health services/ or exp immunization programs/ or exp Immunization/) and (exp Child/ or exp Infant/) | 32499 |
| ((mother* or maternal) adj5 (health* or care or service?)).ti,ab. | 25608 |
| ((child* or infan* or baby or babies or p?ediatric* or preschool* or pre-school* or toddler?) adj5 (health or care or service?)).ti,ab. | 111771 |
| ((reproductive or reproduction or pregnan* or prenatal or pre-natal or antenatal or ante-natal or antepart* or ante-part* or perinat* or postnat* or postpart* or post-part*) adj5 (health* or care or service?)).ti,ab. | 48713 |
| ((prevent* adj5 (health* or care or service?)) and (child* or infan* or baby or babies or p?ediatric* or preschool* or pre-school* or toddler?)).ti,ab. | 9404 |
| ((prevent* adj5 (health* or care or service?)) and (mother* or maternal or female? or women)).ti,ab. | 10020 |
| ((vaccinat* or immuni?ation*) and (child* or infan* or baby or babies or p?ediatric* or preschool* or pre-school* or toddler?)).ti,ab. | 32994 |
| (child* or infan* or baby or babies or p?ediatric* or preschool* or pre-school* or toddler? or mother* or maternal).ti. | 919616 |
| 1 or 2 or 3 or 4 or 5 or 6 or 7 or 8 or 9 or 10 or 11 or 12 or 13 | 1644991 |
| Family Planning Services/ | 22681 |
| birth intervals/ | 1382 |
| exp Contraception/ | 22542 |
| Contraception Behavior/ | 6322 |
| reproductive control agents/ or exp contraceptive agents/ | 62245 |
| exp Contraceptive Devices/ | 20982 |
| ((family or parenthood) adj2 plan*).ti,ab. | 20174 |
| ((birth* or population or fertility or reproduct*) adj control*).ti,ab. | 9403 |
| ((birth* or population or fertility or reproduct*) adj regulat*).ti,ab. | 1106 |
| ((birth* or pregnanc*) adj2 (spacing or interval?)).ti,ab. | 2592 |
| (pregnan* adj5 (prevent* or interrupt*)).ti,ab. | 9656 |
| contracept*.ti,ab. | 56217 |
| condom?.ti,ab. | 15782 |
| cervical cap?.ti,ab. | 236 |
| diaphragm?.ti,ab. | 23450 |
| (vagina* adj2 (sponge* or ring*)).ti,ab. | 869 |
| (spermicid* or spermatocid*).ti,ab. | 1581 |
| ((intrauterine or intra-uterine) adj2 (device? or system? or coil?)).ti,ab. | 6647 |
| (ius or iud).ti,ab. | 6780 |
| (("morning after" or emergency or postcoital or post-coital) adj2 pill?).ti,ab. | 551 |
| ((female? or women or mother? or maternal) adj5 sterili?ation).ti,ab. | 2426 |
| ((male? or men or father? or paternal) adj5 sterili?ation).ti,ab. | 611 |
| sterili?ation.ti. | 8876 |
| ((fallopian or tube? or tubal) adj2 (sterili?ation or ligat*)).ti,ab. | 3021 |
| vasectom*.ti,ab. | 4150 |
| (coitus interruptus or periodic* abstinence or sexual* abstinence).ti,ab. | 900 |
| 15 or 16 or 17 or 18 or 19 or 20 or 21 or 22 or 23 or 24 or 25 or 26 or 27 or 28 or 29 or 30 or 31 or 32 or 33 or 34 or 35 or 36 or 37 or 38 or 39 or 40 | 181057 |
| Employee Incentive Plans/ | 1625 |
| Physician Incentive Plans/ | 2053 |
| reimbursement mechanisms/ or reimbursement, incentive/ | 14025 |
| exp Fee-for-Service Plans/ | 5040 |
| Contract Services/ | 10155 |
| (conditional adj3 (pay* or transfer?)).ti,ab. | 168 |
| (pay* adj3 performance).ti,ab. | 1712 |
| p4p.ti,ab. | 336 |
| (pay* adj3 quality).ti,ab. | 584 |
| fee? for service?.ti,ab. | 3754 |
| ((incentive? or compensatory or reimbursement) adj plan?).ti,ab. | 207 |
| contract service?.ti,ab. | 95 |
| result? based.ti,ab. | 7693 |
| performance based.ti,ab. | 2873 |
| ((result? or performance or output or out put) adj2 (financ* or fund* or pay* or disburs* or fee? or incentive? or initiative? or contract? or aid)).ti,ab. | 7133 |
| ((pay* or money or monetary or economic* or financ* or reimburse*) adj incentive?).ti,ab. | 4409 |
| ((pay* or money or monetary or economic* or financ* or reimburse*) adj reward?).ti,ab. | 1474 |
| ((pay* or money or monetary or economic* or financ* or reimburse*) adj bonus*).ti,ab. | 54 |
| target* pay*.ti,ab. | 37 |
| 42 or 43 or 44 or 45 or 46 or 47 or 48 or 49 or 50 or 51 or 52 or 53 or 54 or 55 or 56 or 57 or 58 or 59 or 60 | 55060 |
| Developing Countries/ or Medically Underserved Area/ | 68819 |
| exp Africa/ or exp "Africa South of the Sahara"/ or exp Asia/ or exp South America/ or exp Latin America/ or exp Central America/ | 845867 |
| (Africa or Caribbean or West Indies or South America or Latin America or Central America).kf,ti,ab,cp. | 104927 |
| (Afghanistan or Albania or Algeria or Angola or American Samoa or Argentina or Armenia or Armenian or Azerbaijan or Bangladesh or Benin or Byelarus or Byelorussian or Belarus or Belorussian or Belorussia or Belize or Bhutan or Bolivia or Bosnia or Herzegovina or Hercegovina or Botswana or Brazil or Brasil or Bulgaria or Burkina Faso or Burkina Fasso or Upper Volta or Burundi or Urundi or Cambodia or Khmer Republic or Kampuchea or Cameroon or Cameroons or Cameron or Camerons or Cape Verde or Central African Republic or Chad or China or Colombia or Comoros or Comoro Islands or Comores or Mayotte or Congo or Zaire or Costa Rica or Cote d'Ivoire or Ivory Coast or Cuba or Djibouti or French Somaliland or Dominica or Dominican Republic or East Timor or East Timur or Timor Leste or Ecuador or Egypt or United Arab Republic or El Salvador or Eritrea or Ethiopia or Fiji or Gabon or Gabonese Republic or Gambia or Gaza or Georgia Republic or Georgian Republic or Ghana or Gold Coast or Grenada or Guatemala or Guinea or Guinea-Bisau or Guam or Guiana or Guyana or Haiti or Honduras or Hungary or India or Maldives or Indonesia or Iran or Iraq or Jamaica or Jordan or Kazakhstan or Kazakh or Kenya or Kiribati or Korea or Kosovo or Kyrgyzstan or Kirghizia or Kyrgyz Republic or Kirghiz or Kirgizstan or Lao PDR or Laos or Lebanon or Lesotho or Basutoland or Liberia or Libya or Macedonia or Madagascar or Malagasy Republic or Malaysia or Malaya or Malay or Sabah or Sarawak or Malawi or Nyasaland or Mali or Marshall Islands or Mauritania or Mauritius or Agalega Islands or Mexico or Micronesia or Middle East or Moldova or Moldovia or Moldovian or Mongolia or Montenegro or Morocco or Ifni or Mozambique or Myanmar or Myanma or Burma or Namibia or Nepal or Netherlands Antilles or Nicaragua or Niger or Nigeria or Pakistan or Palau or Palestine or Panama or Papua New Guinea or Paraguay or Peru or Philippines or Philipines or Phillipines or Phillippines or Romania or Rumania or Roumania or Rwanda or Ruanda or Saint Lucia or St Lucia or Saint Vincent or St Vincent or Grenadines or Samoa or Samoan Islands or Navigator Island or Navigator Islands or Sao Tome or Senegal or Serbia or Seychelles or Sierra Leone or Sri Lanka or Ceylon or Solomon Islands or Somalia or Sudan or Suriname or Surinam or Swaziland or Syria or Principe or South Sudan or Tajikistan or Tadzhikistan or Tadjikistan or Tadzhik or Tanzania or Thailand or Timor-Leste or Togo or Togolese Republic or Tonga or Tunisia or Turkey or Turkmenistan or Turkmen or Tuvalu or Uganda or Ukraine or Uzbekistan or Uzbek or Vanuatu or New Hebrides or Venezuela or Vietnam or Viet Nam or West Bank or Yemen or Zambia or Zimbabwe or Rhodesia).hw,kf,ti,ab,cp. | 1801356 |
| ((developing or less* developed or under developed or underdeveloped or middle income or low* income or underserved or under served or deprived or poor*) adj (countr* or nation? or state? or population? or world)).ti,ab. | 60484 |
| ((developing or less* developed or under developed or underdeveloped or middle income or low* income) adj (economy or economies)).ti,ab. | 289 |
| (low* adj (gdp or gnp or gross domestic or gross national)).ti,ab. | 158 |
| (low adj3 middle adj3 countr*).ti,ab. | 3956 |
| (lmic or lmics or third world or lami countr*).ti,ab. | 3499 |
| transitional countr*.ti,ab. | 110 |
| 62 or 63 or 64 or 65 or 66 or 67 or 68 or 69 or 70 or 71 | 2152237 |
| 41 and 61 and 72 | 174 |
| limit 73 to yr="2004 -Current" | 49 |
| 14 and 61 and 72 | 494 |
| limit 75 to yr="2004 -Current" | 360 |
| 76 not 74 | 327 |
| 61 and 72 | 3695 |
| limit 78 to yr="2004 -Current" | 2583 |
| 79 not (74 or 76) | 2207 |
