# Supplementary material for: Paying for Performance to Improve the Delivery and Uptake of Family Planning in Low and Middle Income Countries: A Systematic Review
Source: Stud Fam Plann. 2016 Nov 17;47(4):309–24. doi: 10.1111/sifp.12001 (PMC5434945; doi:10.1111/sifp.12001)
Supplement: Supplementary file 2 — Appendix Table 2: Primary and secondary outcomes of review [file SIFP-47-309-s002.docx]

**Appendix Table 2: Primary and secondary outcomes of review**

| **Primary Outcomes** | **Secondary outcomes** |
| --- | --- |
| Use of family planning (FP) services  Continuation and switching  New FP users  FP prevalence rate (modern methods, overall and by method)  Unmet need (modern methods)  Changes in methods mix | Cost, efficiency and/or cost-effectiveness  Acceptability/satisfaction of services for clients  Quality and range of care and services  Impacts on service organisation (e.g. integration, management and information systems)  Unintended effects (e.g. unintended behaviours, distortions, cherry-picking/cream-skimming, gaming)  Equity  Financial risk protection  Provider satisfaction/behaviour change  Sustainability or scale up  Fertility changes  Health outcomes (e.g. reduction transmission of STIs, maternal deaths averted) |
