# Supplementary material for: Paying for Performance to Improve the Delivery and Uptake of Family Planning in Low and Middle Income Countries: A Systematic Review
Source: Stud Fam Plann. 2016 Nov 17;47(4):309–24. doi: 10.1111/sifp.12001 (PMC5434945; doi:10.1111/sifp.12001)
Supplement: Supplementary file 3 — Appendix Table 3: Characteristics of included studies: the intervention [file SIFP-47-309-s003.docx]

**Appendix Table 3: Characteristics of included studies: the intervention**

| **Country** | **Study** | **Study design** | **Dates of study data reported** | **Type of intervention** | **Study population** | **Description of P4P intervention** | **Ancillary components and external contextual factors** | **Theory of change** | **Payment regularity** | **Recipient of payment** | **Payment additional to previous revenue** | **Amount received by facility and health workers** | **Process of verification of payment amount due, and by whom** |
| --- | --- | --- | --- | --- | --- | --- | --- | --- | --- | --- | --- | --- | --- |
| Afghanistan |  |  |  |  |  |  |  |  |  |  |  |  |  |
|  | Engineer  2016 | cRCT | 2010-2012 | Research | 442 facilities in 11 (out of 34) selected provinces were stratified, matched, and then randomly allocated to intervention or comparison arm | Payment for unit service and equity of service provision, adjusted for quality | Ancillary components:  Negotiation by NGOs with MOPH to adjust targets and payments according to baseline contextual differences.  External context:  Delivery of Basic Package of Health Services had improved, but coverage and quality still poor for MCH services. Significant involvement of NGOs in service delivery. Previous performance-based contracting. NGOs were contracted to provide services to facilities in an entire district (i.e. both intervention and control facilities).    Both intervention and control facilities in a province were managed by the same NGO.Control facilities did not receive any bonuses. | P4P expected to increase coverage, by improving provider motivation, and communication with patients. Services were expected to become more equitable and higher quality, with greater patient satisfaction. | Quarterly | NGOs in charge of health facilities | Yes | 10% of total pament was retained by NGOs central offices. The remaining amount was distributed by NGOs to health workers.  Bonuses paid to staff were 6-11% above base salary, increasing to 14-28% after complaints were made about the level of bonuses. | Monthly reports submitted by health facilities. Verified quarterly by independent monitors, record matching, and random patient home visits. |
| Burundi |  |  |  |  |  |  |  |  |  |  |  |  |  |
|  | Bonfrer  2014 | CBA | 2006 - 2008 | pilot, then roll out | Intervention: 2 intervention provinces (Bubanza, Cankuzo)  Control: 2 control provinces (Karuzi, Makamba) selected by MoH in terms of comparable income and for-profit health care facilities. | Payment for unit service and quality | External context: user-fees abolished nationally for pregnant women and children under 5y in May 2006. To replace the lost income from eliminated user fees, facilities received payments from the Government for the services provided for free. Ensuring timely payment to the facilities proved problematic.  In April 2010 the MoH incorporated payment for maternal and child health services into the PBF scheme.  Facilities in control provinces did not receive extra cash support. | P4P was expected to improve quality, which would improve patient satisfaction and attendance, which would increase contraception use and lower childbirth. | Quarterly | Health facility | Yes | Total payment to facility calculated as weighted sum of the number of provided services in the previous 3 months x unit payment x quality bonus (ranges between 1 and 1.25). Averaged 40% of facility income | Monthly reports from health facilities to MoH. Verified and validated by provincial committee through unannounced observation visits to facilities. Quality assessed quarterly by local regulatory authorities on a randomly chosen day using checklist. |
|  | Falisse  2014 | CBA | 2006 - 2009 | pilot, then roll-out | Intervention: 3 provinces (Bubanza, Cankuzo, Gitega).  Control: 7 control provinces were matched using control variables, where the P4P initiative was not rolled out until 2010. | Payment for unit service | External context: user-fees abolished nationally for pregnant women and children under 5y in May 2006.  Previous state and international support to health facilities had been based on inputs - usually including rehab and construction of buildings, purchase of new equipment and drugs, and payment of salaries and bonuses. Yet use and quality of services remained very low. | P4P would give an extrinsic motivation for staff, would improve planning and management, and would clarify the roles of different actors. This would improve the overall functioning of the health system, improve quality, and increase coverage. | Unclear | Health facility | Unclear | Payment to health facility for: day-to-day operations (drug purchase, cleaning materials etc.), small investments in equipment and facilities, and financial motivation of health workers.  Authors cite a survey in the discussion which reported an increase in salaries and bonuses of qualified nurses from US$ 75 in 2006 to US$ 262 in 2011 in P4P facilities. | Third-party agency |
| DRC |  |  |  |  |  |  |  |  |  |  |  |  |  |
|  | Huillery  2014 | RCT | 2009 - 2013 | research | Intervention and control: 96 health areas (152 health facilities) in Haut-Katanga province were randomly assigned to P4P or fixed payments | Payment for unit service (relative to other intervention sites – relative prices attached to targeted services were constant, but absolute prices and facility payments were determined by the quantity of services provided by the facility relative to the quantity of services provided by other incentivised health facilities) | Ancillary components:  Intervention: Facilities were paid on achievement of quantity results relative to achievement in other intervention health facilities – facilities had autonomy to allocate payments among facility staff (including non-technical workers not on government payroll).  Control: Same total amount of Government resources was also allocated to control group to neutralise resource effect (this was paid as a fixed bonus to government payroll health workers).  External context:  Poor quality of health services at baseline. Ratio of health workers to population, and accessibility to facilities was good. Poor infrastructure (1 in 4 facilities had access to water tap), and 1 in 4 health workers did not receive fixed wage from the health facility.  Poor health status of population - 25% of sample sick in the last 4 weeks. 31% of births in the last 12m not in a health facility. Only 13% of children <5y able to present an immunisation card. | Extensive conceptual modelling and citation of theories of change based on external vs. intrinsic rewards/ motivators.  New habit-forming based theory of motivation proposed. Based on two assumptions: 1) people have limited attention re: motives (extrinsic or intrinsic), 2) attention is prone to habit-forming. | Monthly | Health facility | Unclear | Financial incentive payment mechanism resulted in a 34% reduction in staff revenues (because facilities reduced user fees to attract patients in the intervention group)  Control facility health staff received fixed bonuses.Distribution amongst staff was more egalitarian in P4P areas (including non-technical staff – 93% received funds, compared to 77% in control districts). Revenues to P4P facilities reduced by 42%. | Monthly reports from health facilities. Verification of submitted reports compared reported volumes with facility registers, and verified facility register accuracy in the community. System of financial sanctions was set, however community verification was weak and there was poor implementation of sanctions.  PBF facilities more likely to fill out consultation reports than in controls – suggesting under-reporting by non-incentivised workers. No significant differences found in propensity to over-reporting. |
|  | Soeters  2011 | CBA | 2005 - 2008 | research | Intervention: Katana and Idjwi districts (population 300,000).  Control: 2 neighbouring districts with similar characteristics targeted as control areas - Kalehe and Kabare (population 232,000) - assistance before during and after study - IRC (DFID) and BCC (World Bank) from 2007 - essential drugs and equipment and fixed staff bonuses. | Payment for unit service and quality | Ancillary components: Intervention: greater autonomy of facility management (including purchasing drugs etc. and subcontracting to private providers), autonomy in the setting of patient user fees, requirement to make business plans, received coaching.  Remote health facilities also benefitted from an isolation bonus of up to 15%. Abolition of informal taxation on health facilities by local health authorities.  Context: 85% increase in per capita annual cash income in overall study area, from $65 in 2005 to $122 in 2008. War ended just before study, followed by years of relative stability, allowing free movement of goods and people. | P4P would motivate health facilities and their staff to improve performance. | Monthly | Health facility | Yes | Monthly subsidies to P4P facilities varied between $200 and $4000 | External consultants (quantitative targets). Patient questionnaires by community groups. Quality scores by district health authority inspections every quarter. |
| Nicaragua |  |  |  |  |  |  |  |  |  |  |  |  |  |
|  | Regalia  2007 | CBA | 2002 - 2004 | national roll out | Intervention: 668 households. P4P strategy was part of phase II of a large programme (RPS – Red de Proteccion Social) which included significant demand-side payments.  Control: 615 households. Control localities for the second phase were selected among those where the RPS did not plan to expand, by matching through statistical techniques | Payment for unit service – initially based on achievement of 98% coverage targets (all or nothing approach), but later paid according to population covered.  (note: substantial demand-side CCT element) | Ancillary components: Intervention: Significant demand-side CCT component (payments made to poorer families fitting specific criteria, for engagement with preventative health care, and attendance at school). Increased number of health services (mobile clinics to remote areas) to achieve compliance with maximum distance targets (an hour walk) from beneficiaries. Involvement of Promotoras (lay women selected by community) in health outreach.  External context: a modernisation project was commenced in 2001, supported by external donors, to reform publicly provided medical care. Management agreements were made. Health facilities had been understaffed and chronically underfunded. | P4P would provide strong incentives for health providers to develop and implement efficient plans to quickly expand service coverage and outreach activities in areas which were significantly underserved. | Every 2nd or 3rd month | Private contracted providers | Unclear (sounds like contractors were not previously employed by MoH) | Providers were paid upfront fee of 3% of the entire amount before provision of preventative health care services. Health care providers sub-contracted health care teams whose members were paid on average 30-50% more than the MoH personnel operating in the same municipalities | Submitted by providers to Red de Proteccion Social (RPS), verified by RPS and independent external auditors every 6m. |
| Rwanda |  |  |  |  |  |  |  |  |  |  |  |  |  |
|  | Gertler  2012 | CBA | 2006 - 2008 | national roll out | Intervention and control districts were initially randomised but assignment was modified after new district boundaries following Government decentralisation  At baseline clinics had approximately 80% of essential drugs and equipment for curative care services, 78% for deliveries, 96% for prenatal care, and 94% for immunisations. Providers knew 66% of protocols but only delivered about 45%. | Payment for unit service and quality | Ancillary components: Intervention: Quality Improvement advice received from district management team during quarterly visits (intervention).  Control: increased funding was given to match the approximate cost of P4P (traditional input-based budgets of control group were increased by the average amount of P4P payments to intervention facilities) | P4P would increase quality and quantity. It would be more effective for services where relative price increase was highest, and had the highest relative marginal return to effort (i.e. the provider had more control). | Quarterly | Health facility | Yes | Overall P4P represented 24.6% increase in funding above base budget. On average 77% of funds used to compensate personnel, resulting in increase of 38% in staff compensation.  Control sites were given extra funding to compensate | Monthly reports from health facilities to steering committee. Steering committee verifies data and authorises payment. Quarterly unannounced auditing visits to facilities. District hospital team visits every quarter to assess quality, and then discusses findings and ways to improve quality. Additional patient survey. |
|  | Lannes  2015  (and Basinga 2011) | CBA | 2006-2008 | national roll out | Started as RCT, but some health centres reassigned following decentralisation.  19 rural districts: 12 intervention, and 9 control.  166 primary healthcare facilities and 2145 households in baseline and follow up surveys | Payment for unit servuce and quality | Ancillary components:  Budgets of control facilities were increased by the average P4P payment to treatment facilities, to control for additional resources in treatment facilities.  Contextual factors:  In addition to P4P, in Rwanda around the time of the study there were increases in workforce and their skills, health insurance, and better leadership and governance.  Rwanda has low corruption, well-grounded performance culture, large coverage of health insurance, rapid and successful implementation of decentralisation.  Large increase in access to family planning for the poorest between 2006 and 2008 (+19%). | P4P encourages effort and compliance with recommended clinical practice, improving the performance of the health service delivery system, and leading to improved access to health services. | (Quarterly) | (Health facility) | Yes | (On average, facilities in the intervention group allocated 77% of the P4P funds to increase personnel compensation, amounting to a 38% increase in staff salaries; facilities in the control group allocated 73% of the additional input-based funds to increase personnel compensation) | (Monthly reports submimtted by health facilities. District steering committee responsible for verification of data and authorisation of payment. Unannounced auditors sent to facilities every 3 months, and unannounced visit by district hospital team every quarter to assess quality score.) |
|  | Meessen  2006 | CBA | 2001 - 2004 | pilot | Intervention: a total of 19 health centres in 2 rural health districts- Kabutare (population est. 302,750 in 2002) and Gakamo (population est. 85090). Previously support in both districts by NGOs. Infrastructures were new and of good quality, few stock-outs, computerised HIS.  Kabutare had sufficient staff, and had pre-existing fixed allowance scheme (which was revised to P4P). Gakoma had difficulties attracting staff (more rural) and no pre-existing allowance scheme.  Patient attendance was declining in real terms - thought to be in part due to user fees, though facilities thought to be of higher quality were seeing more patients. Suggestion of non-acceptance of FP.  Control 1: comparison with selected (matched) facilities from national database before and after the Performance Initiative  Control 2: Cyangugu district - different P4P scheme, and involved community in monitoring. | Payment for unit service | Ancillary components: Intervention: included training sessions, introduction of new protocols, steering committee of key partners, management committee formation, motivation contracts for individual health workers including variable bonus scheme and job descriptions, signing of purchasing contract by health centre, support from Government in timely decisions of posting or transfer of personnel.  External context: "Mutuelles" community health insurance scheme introduced by Government | Facilities already had other institutional determinants in place, so was felt that P4P was the intervention required. Previously fixed bonuses had not led to improved performance. | Monthly | Health facility | Gakoma: Yes  Kabutare: previous fixed individual bonus system | Initiative contributed to up to 39% of the staff income in the health centres in Kabutare district | Monthly reports from health facilities to steering committee. Independent complementary monitoring conducted by School of Public Health of Butare (including home visits to users randomly selected from daily registers). |
|  | Rusa  2009 | ITS | 2004 - 2007 | Pilot, then national roll out | Intervention: 74 health centres in 5 rural districts (Ruli, Rutongo, Kabgayi, Kabuga, Bugesera) and one semi-rural district (Muhima), covering 2,072,282 inhabitants (pilot phase). In 2006, this strategy was extended to an extra group of 85 HCs in other rural districts and so covering a total of 3,784,632 inhabitants (roll-out phase). Those districts had been selected because they were not covered by any other P4P initiatives. Geographical access to HC comparable with other districts of the country. Evidence of progress attained over last 10 yrs. High level of immunisation coverage in 2005 of over 90% DPT 3rd dose vaccine by 1st year of age. Improvements in neonatal and maternal mortality, but still high. Only 9.5% of adult women used contraception in 2005. | Payment for unit service and quality | Ancillary components: Intervention: Supervision, training, and clarification of roles and responsibilities. Financial support for administration, supervision and training was provided in addition.  External context: community health insurance (Mutuelles) adherence increased every year after it was implemented in 2002, other initiatives such as removing delivery fee after 4 ANC standard visits, free baby cloths when delivering in a HC etc. were led by the MoH, national sensitisation campaigns launched by the national media and the civil authorities. Increase in base salaries in 2007. Strengthening of other components of the health system, such as data collection, monitoring, and integrated supervision, involvement of community health workers who were subcontracted by the HC receiving a small bonus for every referred client. | Pilot projects and literature identified P4P as successful in improving productivity or volume of healthcare services and maintaining or improving quality. | Monthly | Health facility | Yes | Monthly average salary of A2 auxiliary nurse in 2005 was 45,000FRW, and in 2007 92,315FRW (US$1 = 555FRW). Average ratio subsidy/salary 39% in 2005, 84% in 2006, 40% 2007. A ceiling of payment was applied in 2005, lifted in 2006, and a rise in salaries in 2007 dropped the ratio.  The majority of health facility payments were used as salary top-up, and some was used for functioning of health facility (8% in 2005, 23% in 2006, 38% in 2007) | Targets measured by health facilities, and then measured again and verified by monthly visits by 2 district supervisors of the MoH, all reports subject to central checking process to look for suspicious reports - systematic re-verification process |
|  | Priedeman Skiles  2013 | RCT | 2005 - 2008 | national roll out | Intervention and Control: Random allocation of paired districts to early or late implementation of P4P during scale up. Matched paired districts. No significant differences in baseline characteristics | Payment for unit service and quality | Ancillary components: Intervention: training, supervision, and accountability through monitoring and reporting of services provided. | P4P would motivate health providers to increase service output and improve quality of care. | Monthly | Health facility | Yes | Average 38% salary top-up. Typically 3/4 of funds were directed to health providers and remainder used for infrastructure and supplies | Gov of Rwanda MoH |
|  | Soeters  2005 | CBA | 2001 - 2004 | pilot | Intervention: 2 public and 2 church based health centres in each of Butare (pop. 380,000) and Cyangugu (pop. 624,000) rural provinces.  Control: 2 public and 2 church based health centres from 2 non-P4P provinces were selected. Gikongoro received more Gov support than other provinces in salary payments. Kibungo benefitted from EU item line financing since late 1990s | Payment for unit service | Ancillary components: Intervention: Differences in implementation of intervention across the different provinces - in Cyangugu province, managers were more proactive in developing FP services, e.g. recruiting additional nurses for community outreach. Different management structures and processes, and different funding. Cyangugu put a lot of effort into multi-stakeholder participation.  Control: control provinces experienced different conditions - one benefited from increased staffing from the Gov, and the other benefitted from an EU project focusing on supervision | P4P would be compared to traditional item line financing, and to an alternative strategy of provision of extra staff. | Unclear | Health facility | Unclear | Butare: some opted for retaining 5% of payment for investing in facility. Cyangugu: 40% given as staff bonus payments, 60% reinvested in facility. HW income in the Gikongoro and Kibungo provinces were 22.7% lower than Butare and Cyangugu. | Butare: data from HIS, monitored by steering committee, third party monitoring by Butare School of Public Health. Cyangugu: supervisors and officer for monitoring and evaluation to validate data and survey patient satisfaction, community organisations. |
| Tanzania |  |  |  |  |  |  |  |  |  |  |  |  |  |
|  | Binyaruka  2015 | CBA | 2012-2013 | evaluation | Intervention: 7 disctricts, 6 hospitals, 16 health centres, 11 faith-based and parastatal dispensaries, 42 public dispensaries.  Control: 4 neighboring comparison districts (Kilwa, Mvomero, Morogoro town, Morogoro rural) – same number of facilities as intervention arm | Payment for unit service and quality of care | Ancillary components: district and regional managers also received bonus payments based on performance of the facilities in their districts.  Providers paid traditional birth attendants for referrals, and extended opening hours, to attract more patients to targeted services.  Context: P4P scheme introduced in 2011 in Pwani region of Tanzania | Financial incentives will make health workers more motivated to deliver quality care and attract patients to the facility. Additional funding also anticioated to improve facility resources and improve quality of services, and perhaps user fees might be reduced to attract more patients. | Every 6 months | Health facility  (and district and regional managers) | Yes | At least 75% of payment should be distributed to health workers, and remainder retained by facility. Maximum payment per cycle:  Dispensaries = US$ 820  Health centres = US$ 3220  Hospitals = US$ 6700.  Health worker payment is equivalent to around 10% of salary.  Distirct and regional managers receive payments of up to US$ 3000 per cycle | Verified each cycle by national, regional and district stakeholders – compare aggregate data to facility registers. |
