# Supplementary material for: Paying for Performance to Improve the Delivery and Uptake of Family Planning in Low and Middle Income Countries: A Systematic Review
Source: Stud Fam Plann. 2016 Nov 17;47(4):309–24. doi: 10.1111/sifp.12001 (PMC5434945; doi:10.1111/sifp.12001)
Supplement: Supplementary file 4 — Appendix Table 4: P4P indicators, evaluation data and main outcomes [file SIFP-47-309-s004.docx]

**Appendix Table 4: P4P indicators, evaluation data and main outcomes**

| Country | Study | P4P incentives to health facilities | Evaluation data | | Results | |
| --- | --- | --- | --- | --- | --- | --- |
|  |  | Targeted services and payments (where available) | Sample size studied | Outcome data source/s | Family planning outcomes (primary outcome of review) | Other main outcomes |
| Afghanistan |  |  |  |  |  |  |
|  | Engineer  2015 | Quantity: 9 MCH output indicators   1. First antenatal care visit - US$ 1.30 (revised to US$ 2.67 in Oct 2011) 2. Second antenatal care visit - US$ 1.30 (revised to US$ 2.67 in Oct 2011) 3. Third antenatal care visit - US$ 1.30 (revised to US$ 2.67 in Oct 2011) 4. Fourth antenatal care visit - US$ 1.30 (revised to US$ 2.67 in Oct 2011) 5. Skilled birth attendance cases - US$ 10.37 (revised to US$ 35.63 in Oct 2011) 6. First postnatal care visit - US$ 1.30 (revised to US$ 2.67 in Oct 2011) 7. Second postnatal care visit - US$ 1.30 (revised to US$ 2.67 in Oct 2011) 8. Pentavalent3 vaccination - US$ 3.00 (not revised) 9. Tuberculosis case detection - US$ 5.00 (not revised)   Equity: Balanced scorecard addressing quality of services, and contraceptive prevalence rates in health facility catchment  Quality: score based on a National Monitoring Checklist  (Individual NGOs negotiated with MOPH to adjust their payments, based on differences in baseline variables) | Intervention:  Household survey – 72 (out of 230) facilities and 3421 households in endline survey.  Facility survey - 81 facilities, 285 health workers and 727 patients in end-line health facility survey  Control:  Household survey – 71 (out of 230) facilities and 3427 in endline survey.  Facility survey - 81 facilities, 285 health workers and 727 patients in end-line health facility survey  Endline data used.  Random sampling (in provinces where safety adequate for data collection) | Monthly reports submitted by health facilities  National Monitoring Checklist, assessed quarterly by independent monitoring team  Health facility surveys using Balanced Scorecard (structured observation, interviews, exit survey)  Household surveys | Table 3  Current use of modern family planning methods (mean endline %):  Intervention 10.7 (95% CI 7.9-12.2)  Control 11.2 (95% CI 8.3-12.9)  Difference – 0.5 (p 0.9) | Table 3 - difference (p value)  At least one antenatal checkup from skilled provider +0.6 (p0.9)  Skilled birth attendant present at latest delivery +5.4 (p0.2)  Potnatal checkup within 42 days of delivery by a skilled provider +0.9 (p0.98)  Children received prentavalent3 vaccination -2.7 (p0.4)  Equity of institutional deliveries (concentration index) (p0.3)  Equity of children’s utilisation of outpatient services (concentration index) (p0.98)  Balanced scorecard indicators (total 20 in 5 domains: client and community, human resources, physical capacity, quality of service provision, management systems):  Significant improvement in 3/20 indicators (in quality of service provision domain). No significant difference in the remaining 17. |
| Burundi |  |  |  |  |  |  |
|  | Bonfrer  2014 | Quantity: total of 23 output indicators, of which only 6 chosen for current study:   1. Antenatal care (new and standard visits) – US$ 0.40 2. Pregnant woman fully immunised – US$ 0.50 3. Bed net distributed – US$ 1.50 4. Child under 1 completely immunised – US$ 1.50 5. **Family planning (new and re-attendants, oral and injectable) – US$ 2.00** 6. Institutional delivery by qualified staff – US$ 2.00   Quality: checklist containing 220 items - bonus of up to 25% | Households randomly selected in each round of data collection:  225 intervention  175 control  Additional survey of 75 health facilities | Cross-sectional household surveys  Monthly reports submitted by health facilities  Unannounced visits by MoH to facilities | Appendix 4 calculated difference of differences of phase I vs phase II 2006-2008):  Modern FP methods: +2%  Child birth in the past 12 months: +1% | Appendix 4 (calculated difference of differences of phase I vs phase II 2006-2008):  Institutional delivery: +45%  More than one antenatal care visit: +7%  More than one tetanus vaccination: +4%  At least one childhood vaccination: +3%  BCG vaccination: +2%  Use of at least one bed net: +3% |
|  | Falisse  2014 | Quantity: at least 42 indicators used by different partners including curative services, reproductive health, preventative health and HIV/AIDS. Only 6 P4P indicators were retained in this study:   1. Number of outpatient visits 2. Antenatal visits 3. Antenatal tetanus vaccination 4. Deliveries at the health facility 5. Vaccination of children (entails 4 indicators) 6. **Fitting of IUDs as a family planning method** | 10 intervention provinces  7 control provinces | Burundi National Health Information System (limited capabilities and not always good quality, therefore evaluation focused on activities whose NHIS series were most complete)  (A second dataset (monthly activity reports from 201 health centres used for P4P payment) was also reported in the paper, but did not include a control group.) | Page 6 –  IUD and malaria treatments do not correlate significantly with P4P. | Page 5 -  Indicators on institutional deliveries and prenatal consultations are only borderline significant (for p <0.1) when controlling for the number of public health facilities, private health facilities, and number of nurses per inhabitant. |
| DRC |  |  |  |  |  |  |
|  | Huillery  2014 | Quantity:   1. New curative consultation - US$ 0.6 2. Institutional delivery - US$ 5 3. Obstetric referral to hospital - US$ 5 4. Full childhood immunisations - US$ 3.5 5. Pre-natal care consultation - US$ 1.2 6. 5th dose of tetanus vaccination - US$ 2 7. **User of modern family planning - US$ 4.5**   (Plus 3 additional targeted services at referral health centres- C-section, blood transfusion, and obstetric referrals to hospitals).  Facility payments were determined by the quantity of services provided by the facility relative to the quantity of services provided by other incentivised health facilities  No payments related to quality. | (taken from appendix table 2):  Intervention: 44 health areas, 60 health facilities, 154 facility staff, 470 patients, 859 households  Control: 43 health areas, 63 health facilities, 178 facility staff, 544 patients, 849 households. | Baseline survey (Sep-Nov 2009), and final survey (Dec 2012-Feb 2013, 4 months after withdrawal of P4P)  Administrative data (monthly reports from health facilities, with verification)  Qualitative data  Attendance spot-checks | No effect of P4P on use of modern family planning: only 5% of women aged 15-49 used a modern contraceptive method | Page 27-  No effect of P4P on utilisation of immunisation  No effect of P4P on institutional delivery, antenatal care visits, postnatal visits, ANC iron supplementation, ANC malaria treatment, or breastfeeding.  P4P had a small negative effect on service utilisation and no impact on delays in seeking care.  No improvement in health outcomes (weight-for-height z score, general mortality rates, maternal mortality, under-5 mortality, or neonatal mortality) |
|  | Soeters  2011 | Quantity:  Health centres received subsidies for 16 indicators, such as:   - Outpatient department consultancies - Number of bed days - Fully immunising a child before 12 months of age - Construction of a household pit latrine - **Use by a women of oral or injectable contraceptives**.   Hospitals received subsidies for 22 general indicators and 8 HIV indicators.  Monthly subsidies to participating facilities varied between $200 and $4000. In addition to the basic subsidies, remote health facilities benefited from an isolation bonus of up to 15%.  Quality:  Quality reviews were also done every 3 months - health facilities received a bonus based on these: up to 15% of the subsidies for health services provided if the score was 100%, and proportionally less for lower scores. | Cluster of 240 households in intervention arm and 200 households in control arm | Stratified household cluster surveys | Page 1522 –  Woman in household using modern family planning 2008/2005 (% change – absolute difference in % coverage, before and after):  Intervention group: 12%.  Control group: 8%.  Difference between groups 4% - ‘statistically weak’. | Page 1522-  Only 2 indicators produced significant results:  In intervention group: whether patient had heard about HIV/AIDS  In control group: childbirth occurred in health facility |
| Nicaragua |  |  |  |  |  |  |
|  | Regalia  2007 | Payment of services provided made on basis of achievement of coverage targets.  Phase II - maternal health **including family planning** (distribution of contraceptives to women of childbearing age and adolescents)  Multiplied number of people served (by different groups) by cost of the specific service provided. | Phase 2: 688 intervention households and 615 control households | Record of service delivered or visit, signed, and kept by provider and by beneficiary. Forms stored by providers and periodically collected by RPS team. This info used to assess household compliance with programme requirements and whether coverage targets achieved by providers.  Random checks by RPS team every 6m - providers, households and individual beneficiaries.  Independent external audit every 6m of random checks of records. | Page 41.  During Phase II the programme generated a net average impact of 5% in the use of FP methods by women aged 12-49 (from initial level of 24%).  The impact was 3 times greater among women 30-40yrs.  The qualitative evaluation stresses the variation across localities with respect to the practice of FP, mainly related to religion, with less support for it in evangelical localities | Page 42.  The RPS net impacts on use of maternal care services were rather modest, mainly due to improvements in the control group. |
| Rwanda |  |  |  |  |  |  |
|  | Gertler  2012 | Visit and outreach indicators:   1. Curative care visits – US$ 0.18 2. First prenatal care visits – US$ 0.09 3. Women who completed 4 prenatal care visits - US$ 0.37 4. **First time family planning visits (new contraceptive users) – US$ 1.83** 5. **One-month contraceptive resupply visits – US$ 0.18** 6. Deliveries in the facility – US$ 4.59 7. Child (0-59m) growth monitoring/ preventative care visits – US$ 0.18   Content of care indicators:   1. Children who completed vaccinations on time – US$ 0.92 2. Appropriate tetanus vaccine during prenatal care – US$ 0.46 3. Second dose of malaria prophylaxis during prenatal care – US$ 0.46 4. Appropriate referral to hospital for delivery – US$ 1.83 5. Appropriate emergency transfers to hospital during delivery – US$ 4.59 6. Malnourished child referrals to hospital during preventative care – US$ 1.83 7. Other emergency referrals during curative treatment – US$ 1.83 | Intervention: 10 districts, 80 primary care facilities  Control: 9 districts, 86 primary care facilities | Monthly reports submitted by health facilities    Face to face interviews - facility survey, household survey  Provider resources, provider skill (knowledge) and quality of care | Table 7 (comparison with controls):  Use of modern contraceptives:  No statistically significant difference seen (p 0.27) | Table 7 (comparison with controls):  Maternal health care utilisation:  Pre-natal care – no significant difference  Institutional delivery – significant increase (p 0.04)  Utilisation by children 0 -47 months:  Preventative care – significant increase (p 0.003. p 0.000)  Curative care visit – no significant difference  Quality of prenatal care:  Tetanus vaccination p 0.07  Clinical protocol Z-score p 0.04 |
|  | Lannes  2015  (and Basinga 2011) | Incentivised services as part of P4P strategy, for which baseline data showed lower utilisation by the poorest:   1. Institutional deliveries (US$ 4.59) 2. Use of modern family planning (first time family planning visit US$ 1.83, woman received 1-month supply of contraceptives US$ 0.18) 3. Four or more antenatal care visits (US$ 0.37) 4. Prenatal care during the first quarter (US$ 0.09) 5. Preventative and curative child care in the past 4 weeks | 19 rural districts: 12 intervention districts and 7 control districts  166 primary health care facilities, and 2145 households in the catchment of these facilities | Baseline and follow-up ousehold surveys (same households) | Table 9  Effect of P4P on modern contrecptive use not significant when looking at whole sample. However, there were significant changes in usage when sample divided by wealth group.  Use of modern contraceptive (uppper wealth group) – difference in difference +17% (p<0.01)  Use of modern contraception (lower wealth group) – difference in difference -10% (not significant)  Effect of P4P on family planning use significantly negatively effected by health insurance in the lower wealth group only. Authors suggest this could be a crowding out effect, or may reflect overrepresentation of pregnant women in the insured population. | Institutional deliveries:  Positive impact of P4P found in upper wealth group only. No impact of P4P in lower wealth group, however if a poor woman had health insurance and lived in catchment of P4P facility, she was 15% higher chance of institutional delivery.  Prenatal care service:  No effect  Child health services: Table 10  Preventative care – significant increase in both wealth groups (p<0.01)  Curative care – no impact |
|  | Meessen  2006 | Quantity:   1. Delivery at health facility - 2500FRW = more than US$ 5 at the start of the scheme (including If transferred to higher-level health facility) 2. Receipt of between 2 and 5 doses of TT (ANC) - 250FRW 3. **Each new subscriber to modern family planning - 1000 FRW** 4. Measles vaccination – 500 FRW 5. Number of new curative cases – 40 FRW per case | Intervention: 19 health centres (4 in Gakoma district and 15 in Kabutare district)  Controls (2 types of control):  1) Health centres without intervention, which had baseline data for 2001 available, had a population profile matching rural intervention districts, had at least 9 months of data available in 2001 and 2004.  2) 22 health centres from Cyangugu province, another site where an output-based approach was started in 2002 | GESIS- Electronic HIS - monthly health centre activity reports sent to district office.  Independent complementary monitoring conducted (including home visits to users randomly selected from daily registers). This checking was done by School of Public Health of Butare | Tables 1-4:  New subscriber to family planning 2004/2001 (% change – proportional change in absolute frequency, before and after):  Control 1 = other rural health centres (+82)  Gakoma v Control 1 : +1323  Kabutare v Control 1: +156  Cyangugu (control 2) v Control 1: +329  Coverage rate for family planning 2004/2001 (% change – absolute difference in % coverage, before and after):  Control 1 = other rural health centres (+253)  Gakoma v Control 1 : -253  Kabutare v Control 1: -97  Cyangugu (control 2) v Control 1: +450 | Tables 1-4 (3 outcomes selected):  Deliveries at health centre 2004/2001 (% change - proportional change in absolute frequency, before and after):  Control 1 = other rural health centres (+53)  Gakoma v Control 1 : +172  Kabutare v Control 1: +103  Cyangugu (control 2) v Control 1: +276  Coverage rate for TT 2-5 2004/2001 (% change – absolute difference in % coverage, before and after):  Control 1 = other rural health centres (+25)  Gakoma v Control 1 : +58  Kabutare v Control 1: +41  Cyangugu (control 2) v Control 1: n/a  Coverage rate for measles immunisation 2004/2001 (% change – absolute difference in % coverage, before and after):  Control 1 = other rural health centres (+3)  Gakoma v Control 1 : +35  Kabutare v Control 1: +4  Cyangugu (control 2) v Control 1: n/a |
|  | Rusa  2009 | Quantitative targets would only achieve payment if specific quality criteria had also been fulfilled:  (US$ 1 = 555 FRW)   1. Curative consultations (total new cases during the month fulfilling quality criteria) – 100 FRW 2. Antenatal consultations (total new and old cases during the month fulfilling quality criteria) – 100 FRW 3. Growth monitoring consultations (all children of >1y and <5y present at consultation fulfilling quality criteria) – 100 FRW 4. **Family planning consultations (Total women protected at the end of the month fulfilling the quality criteria: FP record correctly and completely filled out and correctly classified) - 100FRW** 5. Deliveries (assisted deliveries at the health centre fulfilling the quality criteria, or correctly transferred for delivery at the district hospital) – 2000 FRW 6. Vaccinations (total of children ≤1y completely vaccinated during the month fulfilling quality criteria) – 1000 FRW | 159 health centres | Monthly monitoring visits by district supervisors | Figures 4, 7, 8:  Text pages 833 – 834 reports no impact of strategy on family planning prevalence, but improvements in quality. | Figure 1 and 2:  Positive impact of P4P on institutional deliveries and growth monitoring consultations.  Figures 3-6:  No impact of P4P on quantity of activities found for curative consultations, family planning, antenatal consultations, or vaccinations.  Figures 7 and 8:  Quality of services improved in both groups. |
|  | Priedeman Skiles  2013 | Quantity:  14 indicators for maternal and child health services including, but not limited to: ANC use, delivery in a facility, **modern contraceptive use (pill, injectables, implants or IUD)**, immunisation, growth monitoring and appropriate treatment and referrals  Quality:  9 quality indicators were used to weight payment based on quality score | Panel dataset:  7899 women 15-49yrs of age: 4477 intervention, 3422 control (3611 women from 2005 survey and 4288 from 2007).  Family planning dataset:  4121 currently married women: 2328 from intervention districts and 1793 from control districts | National Demographic Health Survey data | Table 2:  Use of modern contraception 2007/2005 (% change – absolute difference in % coverage, before and after):  Intervention group + 15.9% (p<0.001)  Control group: + 16.5% (p<0.001)  No significant difference between intervention and control groups | Table 2:  Facility deliveries 2007/2005:  Intervention: +36%  Control: +19.9%  All other outcomes (First trimester ANC, Four or more ANC) showed significant improvements in both intervention and control groups, but no significant difference between groups. |
|  | Soeters  2005 | Quantity:  Butare scheme subsidised 6 indicators at health centre level:   1. New consultation – 40 FRW 2. Institutional delivery – 2500 FRW 3. Referred delivery – 2500 FRW 4. Measles vaccination – 500 FRW 5. Tetanus vaccination (2-5) – 250 FRW 6. **New subscriber to oral or injectable family planning – 1000 FRW**   Butare did not include district hospitals in scheme  Cyangugu scheme subsidised 11 indicators at health centre level:   1. New consultation – 150 FRW 2. Institutional delivery – 2000 FRW 3. Referred delivery – 2000 FRW 4. Tetanus vaccination (2-5) – 250 FRW 5. Complete infant vaccination – 1000 FRW 6. **New subscriber to oral or injectable family planning – 1000 FRW** 7. **Previous users of oral or injectable contraception – 750 FRW** 8. **Intrauterine contraceptive device (IUD) – 2500 FRW** 9. **Referral for sterilisation – 3000 FRW** 10. New and standard antenatal consultations – 150 FRW 11. Distribution of mosquito nets – 1000 FRW   Cyangugu also subsidised 8 additional indicators at district hospitals, including obstetric surgery and sterilisation procedures. | 8 intervention health centres  8 control health centres | Health worker survey  Quality survey of each health centre  HMIS output data from the 16 health centres (poor reliability – least unreliable FP indicator is new acceptors rate for oral and injectable contraceptives). | Table 38:  New family planning acceptors:  Change in family planning coverage 2004/2001 (% change – absolute difference in % coverage, before and after):  Intervention:  Butare +1.8%  Cyangugu +3.5%  Overall intervention provinces = +2.8%  Control:  Ginkongoro +0.6%  Kibungo -0.1%  Overall control provinces = +0.2%  Difference in % change Intervention v Control: +2.6% | Table 36:  Outpatient department consultations increased by 147% in intervention group and by 52% in control group  Table 37:  Institutional deliveries increased by 10.9% in intervention provinces, and by 2.9% in control provinces  Table 39:  Text - ‘Small and non-significant’ improvement in measles vaccination coverage in intervention compared to control group |
| Tanzania |  |  |  |  |  |  |
|  | Binyaruka  2015 | Coverage indicators (Table 1):   1. Institutional delivery rate 2. % of mothers attending a facility within 7 days of delivery 3. % of women using long term contraceptives 4. % of children under 1 year received measles vaccine 5. % of children under 1 year who received Penta3 6. % ANC clients on IPT2 7. % HIV+ ANC clients on ART 8. Polio vaccine at birth   Performance targets were set for each indicator, according to baseline coverage in the previous cycle. Payments made if 75% of target achieved. Full payment made if 100% of target achieved, otherwise 50% of total available payment made.  Targets relating to partogram completion, maternal and neonatal death audits, and timely submission of HMIS reports were also introduced. | 7 intervention and 4 control districts. 75 health facilities in each arm (6 hospitals, 16 health centres, 53 dispensaries)  Sampling:  75 facilities per arm  10 patients per facility  1500 households per facility catchment area. | (Baseline and endline surveys)  Household survey  Exit unterviews  Facility survey – month utilisation data from patient registers for targeted and non-targeted services | Table 2:  Use of any family planning (%) – difference in difference -0.7 (not significant) | Table 2 (difference in difference reported):  Institutional delivery rate (%) 8.2 (p0.001)  Institutional delivery rate (%) (public) 6.5 (p0.02)  Postnatal care in facility <7 days (%) 0.6 (not significant)  Children under 1 year received measles vaccine (%) 9.6 (not significant)  Children under 1 year who received Penta3(%) 2.4 (not significant)  At least 2 doses of IPT given during ANC 10.3 (p0.001)  HIV ttreatment during ANC (%) -0.3 (not significant)  Polio vaccine at birth (%) 5.6 (not significant)  Non-targeted services:  Any ANC visit (%) 3.3 (p<0.001)  Four or more ANC visits (%) 3.9 (not significant)  Postnatal care in facility <2m (%) -1.6 (not significant) |
