# Supplementary material for: Paying for Performance to Improve the Delivery and Uptake of Family Planning in Low and Middle Income Countries: A Systematic Review
Source: Stud Fam Plann. 2016 Nov 17;47(4):309–24. doi: 10.1111/sifp.12001 (PMC5434945; doi:10.1111/sifp.12001)
Supplement: Supplementary file 5 — Appendix Table 5: Robustness of included studies [file SIFP-47-309-s005.docx]

**Appendix Table 5: Robustness of included studies**

| **Country** | **Study** | **Type of publication** | **Source of funding of P4P** | **Implementing partner/s** | **Unit of allocation** | **Unit of data collection** | **Unit of analysis (and adjustment for clustering)** | **Baseline population coverage (family planning)** | **Setting of incentive targets by whom** | **Selection of reported indicators** | **Risk of bias*** | | | | | | | | | **Remarks of authors on interpretation and generalisability of findings** | **Remarks of reviewers on study design or robustness of findings** |
| --- | --- | --- | --- | --- | --- | --- | --- | --- | --- | --- | --- | --- | --- | --- | --- | --- | --- | --- | --- | --- | --- |
|  |  |  |  |  |  |  |  |  |  |  | **1** | **2** | **3** | **4** | **5** | **6** | **7** | **8** | **9** |  |  |
| Afghanistan |  |  |  |  |  |  |  |  |  |  |  |  |  |  |  |  |  |  |  |  |  |
|  | Engineer  2016 | Peer-reviewed journal | Gov of Afghanistan  World Bank | Government, NGOs | Health facility | Health facility  Household | Intervention v control (no adjustment) | Not reported | Negotiation between MOPH and NGOs managing health facilities | Identified before the trial to represent important health services related to MDGs 4 and 5 | ? | ? | ? | - | ? | + | ? | ? | ? | ...the more relevant question is not whether P4P programmes work, but what circumstances and design characteristics may impact on their effectiveness. The minimal effects found in our study point to some of the pitfalls in the design and implementation of the P4P programmes, consistent with the emerging evidence from other studies.  P4P may be effective in some places, but does not replace the critical need to enhance the broader leadership and management capabilities of health services organisations and to understand and address issues concerning demand for services and barriers faced by communities and households. | Intervention and control facilities in the same province were managed by the same NGO. NGOs were contracted to manage provincial health services by the Government and had negotiating power with MOPH re: performance payments and indicators.  Many health workers did not realise they had received perfoamnce payments. |
| Burundi |  |  |  |  |  |  |  |  |  |  |  |  |  |  |  |  |  |  |  |  |  |
|  | Bonfrer  2014 | Peer-reviewed journal | Gov of Burundi 52%, World Bank 28%, other donors 20% | Government, NGOs | Province | Health facility  Household | Phase I v Phase II  (no adjustment calculation reported) | Modern family planning:  Intervention sites: 9%  Control sites: 9% | Unclear – suggested to be Government and NGOs | Time and financial constraints limited data collection to the chosen indicators | + | + | - | - | + | + | ? | ? | + | Difficult to unpick relative effects of extra resources vs incentives | Confounding variables – P4P started at the same time when user fees were abolished for pregnant women and children under 5 years  Control provinces, unlike intervention provinces, were not given additional resources  Nationwide clashes between government forces and national liberation forces – ceasefire May 2008 |
|  | Falisse  2014 | Peer-reviewed journal | EU, World Bank | Government, NGOs | Province | Province | Intervention v control  (no adjustment) | IUDs (per 10,000 inhabitants per year):  Intervention sites (mean): 21.22  Control sites (mean): 5.12 | Unclear – suggested to be Government and NGOs | Evaluation focused on activities: 1) whose NHIS was most complete, 2) which are the core business of health facilities, 3) which are mainly indicators contracted under P4P scheme | + | + | - | - | + | + | ? | ? | + | Context of selective free healthcare. Unknown interaction or synergy between the two strategies (felt by the authors to be significant). P4P seen to be effective in indicators that were free, and not in those that required user payments. Need to consider P4P as part of the larger health system.  Malaria results for control areas may have been influenced by higher altitude in these areas and lower malaria exposure. | Confounding variables – P4P started at the same time when user fees were abolished for pregnant women and children under 5 years  Three of the authors work on P4P projects in Burundi for Cordaid, and Cordaid (the P4P implementer) funded part of the study.  Authors note that data may not have been of prime quality. |
| DRC |  |  |  |  |  |  |  |  |  |  |  |  |  |  |  |  |  |  |  |  |  |
|  | Huillery  2014 | Online publication | Government of DRC, World Bank | Health Authorities of Haut-Katanga and the Health Sector Rehabilitation and Support project (Projet d'Appui à la Réhabilitation du Secteur de la Santé - PARSS) in Lubumbahi and Kinshasa | Health area | Heath facility  Household | Intervention v control (adjusted for clustering by health area) | Modern family planning:  Overall prevalence cited: 5% of women aged 15-49 | Unclear | Unclear | ? | ? | - | - | ? | + | ? | ? | ? | P4P led to increased effort to attract patients however this did not lead to increased utilisation. Decreases in user fees were not sufficient to increase demand; people sensitive to service quality. This challenges idea that demand for health services is elastic in all contexts.Authors comment on low levels of education and knowledge about modern medicine in this context. Also still partially conflict-affected.  Workers decreased their effort after incentives were removed – an effect not attributed to loss of income but loss of intrinsic motivation. Authors suggest P4P suited to simple tasks. Also note that staff in control areas already incentivised by sharing user fees. | Uniquely looks at data post-withdrawal of P4P supply-side initiative. |
|  | Soeters  2011 | Peer-reviewed journal | Government of DRC, World Bank | Government, NGOs | District | Household | Intervention v control (no adjustment) | Women in household using modern family planning:  Intervention and control: 1% | Local Health Authority and NGOs | Most chosen before analysis, some chosen post-hoc. Methodology reviewed and approved by external researchers. | + | + | ? | ? | ? | + | + | ? | + | There were additional contextual factors such as NGO involvement in control districts (though frequent drug stock outs), and user fee management and informal taxation etc. | Significant involvement of other NGOs in control provinces. P4P evaluation was conducted by individuals who designed the initiative.  Baseline and post-intervention surveys conducted during different seasons.  85% increase in per capita annual cash income in overall study area, from $65 in 2005 to $122 in 2008. War ended just before study, followed by years of relative stability, allowing free movement of goods and people.  Design prone to selection bias. |
| Nicaragua |  |  |  |  |  |  |  |  |  |  |  |  |  |  |  |  |  |  |  |  |  |
|  | Regalia  2007 | Centre for Global Development report | Inter-American Development Bank | Government | Household | Household | Intervention v control (no adjustment) | Use of family planning methods by women aged 12-49:  Intervention: 24% | RPS scheme and government | Unclear | + | + | ? | ? | ? | + | ? | ? | + | Combination of demand and supply side interventions made a difference - this continued even after demand side interventions were withdrawn. | Significant demand-side CCT component. |
| Rwanda |  |  |  |  |  |  |  |  |  |  |  |  |  |  |  |  |  |  |  |  |  |
|  | Gertler  2012 | World Bank report | Gov of Rwanda, World Bank | Government | District | Primary care facility | Intervention v control (adjusted at district level) | Modern family planning:  Intervention and control: 10% | Ministry of Health | Utilisation measures chosen to conform as closely as possible to paid indicators. Childhood indicators split by age group due to expected higher utilisation in younger children, and length of time under the P4P programme (health outcomes). | + | + | - | - | ? | + | ? | ? | ? | Improved access to higher quality care that resulted in substantial improvements in child health outcomes. Provider incentives led to 20% improvement in efficiency. Better results with higher incentives that required lower effort on the part of the provider. Evidence of complementarity between P4P and knowledge of health workers. | Increased funding was given to control facilities to try to isolate incentive effect from the effect of overall resources. |
|  | Lannes  2015  (and Basinga 2011) | Peer-reviewed journal | Unclear | (Government) | District | Household | Intervention v control (robustness checks performed for multiple variables) | Use of modern family planning: Intervention and control: 11% | (Ministry of Health) | Incentivised services | + | + | ? | - | ? | + | ? | ? | ? | PBF is not inherently pro-poor. Its effect on improving the welfare of the poor depends on its design, and the equity concern needs to be built early in the design of the program. Also PBF is unlikely to be the soole mechanism and is likely to be more effective if used in synergy with other programs such as health insurance or selected free healthcare. | Uniquely analyses effects of P4P by wealth group, to look for effects on equity. |
|  | Meessen  2006 | Peer-reviewed journal | Swedish International Development Agency (SIDA) | Government, NGOs | District | Health centre | Results separately presented by district (no adjustment for health centre) | Coverage rate for family planning:  Intervention sites: 0.3% - 0.7%  Control sites:  0.6% | Unclear – suggested to be Ministry of Health and NGOs | Unclear | + | + | + | + | ? | + | ? | + | ? | Significant contextual elements have contributed. The country is very well administered. Reasons to suggest no excessive opportunism from staff. The holistic view of institutional arrangements is important in interpreting findings, to prevent readers misinterpreting lessons and wrongly inferring the suitability of possible replication in totally different institutional environments. | There was marked difference in the organisational structures, implementation of P4P, and outcomes between the two intervention provinces. Each has input from a different NGO.  Authors were promoters of the intervention.  Incomplete data for intervention districts. |
|  | Rusa  2009 | Peer-reviewed journal | Gov of Rwanda, Belgian Technical Cooperation | Government, NGOs | District | Health centre | ITS design. Intervention only (no adjustment) | Monthly contraceptive rate of women of reproductive age:  Intervention sites: 3% - 3.5% (estimate from figure) | Ministry of Health and NGO | Unclear | Interrupted time series analysis:  Intervention independent: +  Intervention effect pre-specified: ?  Intervention affect data collection: -  Allocation concealed: +  Incomplete outcome data: ?  Selective outcome reporting: ?  Other risks of bias: + | | | | | | | | | It is difficult to separate the effects related to P4P from other effects. PBF showed limited impact on volume of services, however large improvements on quality of healthcare services were observed.  The discussion reads: “However, the factor that enabled health centres to score better in volume increase through P4P might have been a better financial accessibility through the reduction of user fees by health facilities and through the start-up of community health insurances. In our study, the only quantity increase through P4P was for observed for activities, which were previously less well organised, child growth monitoring services and institutional deliveries. For both activities, the involvement of community health workers, who were subcontracted by the HC receiving a small bonus for every referred client, played an important role” | Significant ancillary components to intervention. Community health insurance scheme was operational. |
|  | Priedeman Skiles  2013 | Peer-reviewed journal | Gov of Rwanda, World Bank | Government | District | Patient | Intervention v control (adjustment model used in analysis) | Modern family planning (pill, injectable, implant, IUD):  Intervention sites: 6.1%  Control sites: 6.8% (least poor women had the highest utilisation, and the most poor the lowest, in both groups) | Government of Rwanda | Unclear | ? | ? | + | - | ? | + | ? | ? | ? | Overall services may be improved by P4P if baseline use of services is low. | Significant ancillary components. Community health insurance scheme |
|  | Soeters  2005 | World Bank report | SIDA, Cyangugu province Community Development Funds, World Bank, UNFPA. | Government, NGOs - Health Net Int, CORDAID | Province | Health centre | Results presented by province (unclear if overall results for intervention and control adjusted – no details given) | Family planning coverage rate (new acceptors):  Intervention sites: 1.1%  Control sites: 0.3% | NGOs | Choice of family planning indicator limited by quality of HMIS data – new acceptors rate for oral and injectable methods was most reliable, which excludes IUDs, implants and sterilisation. | + | + | ? | + | + | + | ? | + | + | Findings should be interpreted carefully however the global picture suggests that P4P is a promising track for Rwanda. | Findings limited by significant biases. Community nurses were used for FP in one district. Data collection was conducted at baseline and end in different seasons.  Researchers were selected because of their involvement in developing the P4P schemes. Interviewers conducting the surveys were also involved in the schemes.  Retrospective analysis. Some of results based on opinions. |
| Tanzania |  |  |  |  |  |  |  |  |  |  |  |  |  |  |  |  |  |  |  |  |  |
|  | Binyaruka  2015 | Peer-reviewed journal | Gov of Tanzania - unclear | Government | District | Facility  Household | Intervention v control (adjusted) | Use of any family planning: Intervention sites: 36.7%  Control sites: 39.2% | Government of Tanzania | Indicators chosen to represent: targeted services, non-targeted services, and services closely related to targeted services. | + | + | - | + | ? | + | ? | - | ? | Ours is one of the only studies to report effects of P4P on PNC, but there was no evidence of an effect, possibly because cultural barriers may prevent women from seeking care so early after delivery.  The fact tat no effect was detected for four or more ANC visits suggests that spillover effects are restricted to services closely related to the target.  The significant reduction in outpatients visits at dispensaries in our study is consistent with concerns on the deterioration of non-targeted services reported elsewhere.  Given these mixed findings, the net effect of P4P on service utilisation is unclear.  While P4P achieved limited effects on targeted maternal and child health services, overall progress towards universal health coverage was mixed. | Modifications to HMIS introduced alongside P4P system. District and regional managers also received significant bonus performance payments. Difference in religion and socioeconomic characteristics between intervention and control sites. |

*Risk of bias score: 1) Random sequence generation, 2) Allocation concealment, 3) Baseline outcomes similar? 4) Baseline characteristics similar? 5) Incomplete outcome data addressed? 6) Knowledge of allocated intervention adequately prevented? 7) Protection against contamination? 8) Free from selective outcome reporting? 9) Free from other risks of bias?

+ = high risk of bias; - = low risk of bias; ?= unclear risk of bias
