# Supplementary material for: Paying for Performance to Improve the Delivery and Uptake of Family Planning in Low and Middle Income Countries: A Systematic Review
Source: Stud Fam Plann. 2016 Nov 17;47(4):309–24. doi: 10.1111/sifp.12001 (PMC5434945; doi:10.1111/sifp.12001)
Supplement: Supplementary file 6 — Appendix Table 6: Secondary outcomes [file SIFP-47-309-s006.docx]

**Appendix Table 6: Secondary outcomes**

| **Country** | **Study** | **Overall cost of P4P** | **Patient satisfaction** | **Quality** | **Changes to organisational services, or autonomy** | **Unintended effects** | **Patient equity measures** | **User fees** | **Financial risk protection** | **Provider satisfaction/ response/ behaviour** | **Sustainability or scale-up** | **List explicit recommendations for future research** |
| --- | --- | --- | --- | --- | --- | --- | --- | --- | --- | --- | --- | --- |
| Afghanistan |  |  |  |  |  |  |  |  |  |  |  |  |
|  | Engineer  2015 | Not stated | No impact of P4P on client satistfaction | Three measures of service quality showed significant improvement: time with patients, more complete history and examination, and counselling patients | Implementing NGOs were able to negotiate with the MOPH to adjust their payments.  10% of payment kept by NGO office. | Only 37.9% of health workers at P4P sites recognised that they had received any payment from the P4P intervention, although 86.7% reported that the health facility had received performance payments.  Lack of communication to health workers about payment, or performance.  A communication from health workers to MOPH in late 2011 prompted an increase in payments. | No effect of P4P on equity of care use. Wealthier women were more likely to have institutional deliveries than poorer women. |  |  | No difference in motivation or job satisfaction scores. |  | Future research is needed to understand how health care organisations can overcome barriers to utilisation of health services and promote healthy behaviour. |
| Burundi |  |  |  |  |  |  |  |  |  |  |  |  |
|  | Bonfrer  2014 | Not stated but average revenues for health facilities  per person per year increased from US$0.53  to US$2.49 between 2006 and 2010 in Burundi—  an almost fivefold increase | No significant change in patient-reported quality | Pooled overall facility quality score increased by 17% points, representing a relative change of 45% (p0.06)  Appendix 4:  Change in family planning quality score between 2006 and 2008:  Intervention:+18%  Control: -11% | The control provinces (unlike P4P provinces) were not given additional resources |  | No strong evidence of differential effects of P4P across socio-economic groups. | User-fees abolished nationally for pregnant women and children under 5y in May 2006. |  |  | In 2014, P4P had been implemented in almost 700 health facilities. As P4P programme developed and expanded: Payment function was changed from NGO to government  administration. Provincial verification and validation committees (public-private partnerships) were developed to perform contracting and verification functions.  Additional incentives for serving poorer populations.  National strategy – Government committed to allocate 1.4% of annual budget to P4P and related health financing strategies. | Exploration of the relative contributions of subcomponents of P4P programmes - such as targeting of the vulnerable, and engagement of the community.  Effects of P4P on maternal and child mortality.  Whether P4P mainly affects health care use and quality through expanded facility resources or through a change in provider incentives - essential know whether P4P is a cost-effective intervention and whether its effects outweigh its additional administrative burden. |
|  | Falisse  2014 | Between Euro 1100 and Euro 2000 per health facility per month, inclusive of all administrative overheads, supervision and verification costs. |  |  | Growth of P4P indicators was associated with greater heterogeneity in performance between intervention health centres, reflected in standard deviations of indicator measures increasing over time. P4P provinces attracted more qualified staff than control provinces | Unknown interactions and synergies with the free healthcare for pregnant women and children under 5y (felt by the authors to be significant).  P4P provinces attracted more qualified staff than control provinces, but this levelled out as P4P was rolled out nationally | No evidence of diminished inequalities between urban and rural health facilities. | User-fees abolished nationally for pregnant women and children under 5y in May 2006. |  | Authors comment in discussion that P4P provinces attract more staff than control provinces (up to point of national roll-out in 2010) | Scale-up to whole country in April 2010  Authors comment on unknown sustainability of the incentive mechanism itself, and the financing of the scheme. | More empirical research is needed to understand the sustainability of (the incentive mechanism of) P4P and the interaction between P4P and other health policies.  The issue of gaming needs to be explored in the context of low-income countries.  Improvements in tools available for thorough impact evaluations |
| DRC |  |  |  |  |  |  |  |  |  |  |  |  |
|  | Huillery  2014 | Budget was estimated at US$ 0.43/cap/yr (average monthly facility payments were US$ 550 and the average catchment area population was 12,900) | No impact of P4P on patient satisfaction. No significant difference in utilisation of health facilities. Lower patient undertanding of drugs prescribed in P4P group. | Overall results suggested that P4P had no effect on technical quality, which remained low.  Significant negative impact of P4P on quantity and quality of equipment and infrastructure. | P4P facilities had autonomy over payment allocations to staff.  Health workers made more effort to attract patients in P4P group: were present more often, user fees for targeted services were significantly reduced, significantly more preventative health sessions were organised, and more community based outreach (but not statistically significant).  Several unskilled private practitioners were not subcontracted, so forced to stop practicing. | Positive effect of P4P on preventative sessions was concentrated on targeted services but did not happen at the expense of non-targeted services. Health workers salaries became significantly lower in P4P group. User fees were reduced in P4P group to attract patients, and could not be easily raised again. Significant negative impact of P4P on quantity and quality of equipment and infrastructure. Decreased job satisfaction in P4P group. |  | User fees for targeted services were significantly reduced in the P4P group to attract patients, and could not be easily raised again.  Patient payments for drugs also reduced in P4P group. |  | 14% reduction in staff satisfaction in P4P facilities – not clear if related to reduced income, increased effort or exposure to motivation itself. Lower perceived workloads in P4P areas. PBF also increased the proportion of facility heads who worry about the volatility of payments but did not increase the level of conflict within facilities. Staff attendance in intervention facilities was lower than in control facilities when P4P was withdrawn (though was higher when P4P was in place).  Decreased job satisfaction in P4P group compared to control, after withdrawal of P4P.  Previously incentivised health workers were found to attach more importance to material benefits (external motivators) than control health workers. | P4P programme was withdrawn in September 2012 (started in June 2010) | None explicitly stated |
|  | Soeters  2011 | External assistance of approximately $2/cap/yr in intervention and $9-12/cap/yr in control health districts | Intervention group: increase in patient perceived quality composite score from 2005-2008 was 8%. Control group: decrease in patient perceived quality composite score from 2005-2008 was 17%. Difference statistically significant. Greater availability of essential drugs in P4P facilities. |  | Contracting, user fees negotiation permitted in P4P facilities, recruitment of new staff by health managers in intervention facilities, collaboration with private dispensaries through subcontracts.  Health facility income increased from external donors (greater increase per capita in P4P facilities; 36cent increase (from 0) vs 14cent increase (from 17) in control – per capita revenues in 2008 $1.04 in P4P facilities and $0.45 in control facilities. | Qualified staff in P4P health centres increased by 23% from 2005 to 2008, compared to 8% in control sites.  Negotiated incentives of around $2000 per month per district health team in the P4P districts stopped the practice of collecting informal health facility taxes, and encouraged them to concentrate on regulatory and supervisory roles. |  | In P4P health facilities, annual per capita revenues from patient user fees increased by 25% between 2005 and 2008. In control facilities this fell by 43%, because the user fee payments were fixed at a very low level by health authorities. In 2008, P4P centres collected 64 cents per capita from user fees and control centres collected 12 cents. | More spending by patients in intervention than control but better perceived quality, and no catastrophic spending in 2008 in P4P group (compared to 6 households in 2005 in P4P group – no data for control group). | Health centre managers more satisfied with health authority supervision in intervention than control districts. | P4P districts received less external foreign financial assistance than control districts. | How P4P should support the poor. How to expand P4P in different contexts, possibly including sectors other than health. |
| Nicaragua |  |  |  |  |  |  |  |  |  |  |  |  |
|  | Regalia  2007 | Total project phase II US$22 million over 3 years - unable to disaggregate P4P from demand side. |  |  | Parallel changes in organisation structures of MoH. Revision of budget allocation mechanisms to health centres. New concept of sub-contracting to private providers. Outreach clinics. The operation of the RPS programme was negatively affected by excessive centralisation of decision-making process and weak ministerial planning capacity (and frequent changes of ministerial post). |  | Improved access for communities targeted by project (e.g. recipients of CCT programme)  Variation of family planning across localities related to religion, with less support in evangelical localities (qualitative evaluation). | CCTs to poor families fulfilling eligibility criteria, which paid for direct and opportunity costs accrued. CCTs were paid for 3 years, and were reduced in amount each year. |  | Outreach effort – Promotoras (lay women chosen by community) organised groups of women and siblings to attend health check-ups.  Some providers used some of their resources to mobilise school teachers and community leaders, and buy radio air-time to promote services. | P4P programme was not continued beyond external donor funded timescale. | Relative contributions of supply vs demand incentives |
| Rwanda |  |  |  |  |  |  |  |  |  |  |  |  |
|  | Gertler  2012 |  |  |  | Restructuring of health districts, changes in management structures brought about by P4P and the associated data collection and accountability. | Qualitative evidence suggested that health facilities paid community health workers to locate women in late stages of pregnancy and encourage and assist them to deliver at the facility (high P4P fee). Similar collaboration with community health workers for infants and small children. |  |  |  | Qualitative evidence suggested that health facilities paid community health workers to locate women in late stages of pregnancy and encourage and assist them to deliver at the facility (high P4P fee). Similar collaboration with community health workers for infants and small children. Authors concluded that health workers did not put so much effort into attracting patients to targeted services that already had high coverage for example (high effort required for minimal gain).Know-do gap reduced in P4P areas, and evidence of higher returns for more knowledgeable staff. Estimate of 20% gain in efficiency overall (not FP-specific). |  | The effect of conditional cash transfers for increasing utilisation of services that are more in the patient's control |
|  | Lannes  2015  (and Basinga 2011) | (Administrative costs asseociated with P4P US$ 0.3 per capita) |  |  | Budgets of control facilities were increased by the average P4P payment to treatment facilities, to control for additional resources in treatment facilities. |  | The study was conducted in rural Rwanda where the population was mostly poor, compared to the overall population. The effect of P4P did not play equally on different income groups – the wealthier group showed greater benefit.  Table 9  Effect of P4P on modern contrecptive use not significant when looking at whole sample. However, there were significant changes in usage when sample divided by wealth group.  Use of modern contraceptive (uppper wealth group) – difference in difference +17% (p<0.01)  Use of modern contraception (lower wealth group) – difference in difference -10% (not significant) | There was a negative interaction between P4P and health insurance in the case of family planning for the poorest group. Authors suggest this reveals existence of competing interventions (e.g. free contraceptives) and that insured women may not need family planning (i.e. are pregnant). |  |  |  | This peper contributes to a call for more rigorous research to capture unobserved heterogeneity by wealth group.  Further research is necessary to test various designs and models of interations with other financing programmes, in different contexts. |
|  | Meessen  2006 | Yearly cost was around $93,000 (or US$0.24/cap/yr) for 3yrs, distributed as: 62% for incentives to health centres, 27% for incentives to district and province managers, 11% for transaction costs (i.e. Steering committee meetings and 2 surveys by school of public health). As the NGO was already paying fixed bonuses in Kabutare district in 2001 (around US$59,000 per year), one can consider that the incremental improvements cost around US$0.035/cap/yr for 2003) |  |  | Included training sessions, introduction of new protocols, steering committee of key partners, management committee formation, motivation contracts for individual health workers including variable bonus scheme and job descriptions, signing of purchasing contract by health centre, support from Government in timely decisions of posting or transfer of personnel. |  |  |  |  |  | Enthusiasm of those involved in the scheme (more than any scientific proof) has convinced stakeholders to develop strategy in other locations | More attention to institutional arrangements. Greater alliances between operational agencies and scientists need to be built. |
|  | Rusa  2009 | $0.2/cap/year 2005-2007 (2005 BTC, 2006-7 MoH). Additional $0.05/cap/year spent by BTC from 2005-2007 for admin supervision and training. |  | Improved quality of services in intervention and control groups | Supervision, training, and clarification of roles and responsibilities. Also data reporting, supervision visits, some revenue going to health centre, and central monitoring. | Some over-reporting of cases was seen in first months of P4P, but due to errors in monitoring, rather than real abuse. |  | Reduction in user fees by health facilities | Existing community health insurance scheme that was promoted at same time as P4P, as were community incentive programmes for deliveries etc. | Subcontracting of community health workers (incentivised) to refer patients for child growth monitoring and institutional delivery | Scaled up in 2006 | Cost effectiveness, compared with other quality improvement initiatives |
|  | Priedeman Skiles  2013 |  |  |  | Facilities had authority to allocate incentive payments according to perceived need: provider bonuses, equipment, outreach activities. |  | No difference in effect of P4P by wealth or residence (rural/urban) | Benefits of community health insurance scheme cover a standard package of MCH services (including family planning). | Community-based health insurance reached estimated 73% coverage in 2006. Enrolment fee and annual premium (poorest eligible for subsidies) | Anecdotally some facilities used outreach to encourage use by women from poorer households, including waiving or reducing fees, offering transportation, and enlisting community health workers to refer women for services. |  | Explore benefits to equity of a recently adopted community-P4P project |
|  | Soeters  2005 | Butare: $0.24/cap/yr. Cyangugu: $2.00/cap/yr |  | Intervention group outperformed control group with a composite quality score of 75% v 47% (though no baseline data – data collected by cross-sectional survey in 2004) | Contracting and subcontracting in Cyangugu province. Different approach taken in each of the intervention provinces.  Supervision and internal technical meetings felt to be much more common in intervention provinces.  Managers were more proactive in Cyngugu and recruited nurses for outreach. |  |  |  |  | Greater satisfaction in intervention provinces, though variable satisfaction found in health worker survey.  Facilities in Cyangugu province had more autonomy when spending subsidies. Money was used for maintenance, staff training, construction, sub-contracting of private dispensaries, opening of health posts, investments in infrastructure, and employing more staff.  Authors feel that dynamism of team in Cyangugu attracted additional donor funding. | Authors recommend scaling up. Authors remind that once target achievement improves at health centre level, funding must also increase to keep up with increased volume of service provision. | Unknown how reduction in financial contribution by users was distributed among population. Different strategies used to boost contraceptive use in Cyangugu province should be documented, as well as collaboration with private sector. Need to learn about coordination mechanisms in context of health services and also national administrative reforms. 1) What are best mechanisms to ensure that P4P contributes positively to quality of care? 2) Should the subsidy structure be different for activities with 'natural' different coverage rates? 3) What are the relevant indicators for complex services? 4) How many staff are required to operate a fund holder organisation? |
| Tanzania |  |  |  |  |  |  |  |  |  |  |  |  |
|  | Binyaruka  2015 |  | No effect on patient satisfaction for targeted services, but significant improvement in satisfaction for non-targeted services (though there was a reduction in the use of non-targeted services at dispensaries) |  | Modifications to HMIS were introduced alongside P4P in intervention facilities. Payments made based on completeness and timeliness of reports.  Providers paid traditional birth attendants for referrals, and extended opening hours, to attract more patients to targeted services.  Providers spent 17% of their time each month on data generation and verication activities linked to P4P. | Significant reduction in outpatient visits at intervention dispensaries (which represent the majority of health facilities in the region) | No significant differences in effect of P4P on different socioeconomic groups | P4P was associated with greater enforcement of exemptions for delivery care at public facilities. No effect on exemptions for ANC or PNC, nor average amount paid or provision of gifts for these services. |  | Providers paid traditional birth attendants for referrals, and extended opening hours, to attract more patients to targeted services.  Providers spent 17% of their time each month on data generation and verication activities linked to P4P. |  | To identify the optimal time to measure the effects of behavioural change interventions, such as P4P.  Further research on distributional effects, effects on non-target service use and quality, and financial protection is urgently needed, to determine whether P4P will help achieve progress towards UHC or undermine it. |
