# Supplementary material for: Paying for Performance to Improve the Delivery and Uptake of Family Planning in Low and Middle Income Countries: A Systematic Review
Source: Stud Fam Plann. 2016 Nov 17;47(4):309–24. doi: 10.1111/sifp.12001 (PMC5434945; doi:10.1111/sifp.12001)
Supplement: Supplementary file 7 — Appendix Table 7: Excluded studies [file SIFP-47-309-s007.docx]

**Appendix Table 7: Excluded studies**

| **Country** | **Study/ report** | **Reason for exclusion** | **Study population and context** | **Study design** | **Description of intervention** | **Ancillary components** | **Contraceptive method or target outcome** | **Incentive targets** | **Submission of data for payment, and verification** | **Source of funding of P4P** |
| --- | --- | --- | --- | --- | --- | --- | --- | --- | --- | --- |
| Bangladesh |  |  |  |  |  |  |  |  |  |  |
|  | Rahman  2011 | No FP data from control group | Intervention: 12 public-sector health facilities in 3 districts Control: 3 facilities in 1 district.  Utilisation of FP is high in Bangladesh, but low utilisation of long-term methods, high levels of discontinuation. Contraceptive prevalence 61% (72% = replacement level fertility) | CBA | 1) Combination of P4P for providers and demand-side cash transfers for clients  2) P4P for providers only | Development of guidelines and manuals  Formation of PBF committees, Quality Assurance Groups, Quality Assurance Teams, fund operations teams, and MNCH service provider teams. (Coupon mechanism demand-side incentive program for poor clients in one arm) | FP counselling | Quarterly quantitative targets set based on the individual facility's performance. Weighted quality score | Facility reports of service volumes verified by external audit firm | Population council, UNICEF |
| Burundi |  |  |  |  |  |  |  |  |  |  |
|  | Busogoro  2010 | Reports ITS data for Kibuye province on FP - but data given is absolute numbers of women rather than proportions, and in graphical form only | Kibuye district 127,644 inhabitants P4P project managed by HealthNet TPO. | ITS | In the pilot areas managed by HealthNet TPO, health facilities receive 2 types of payment: 1 based on quantitative measurements and the other on qualitative measurements. The payments were linked. |  | IUDs, new and recurrent users, implants, sterilisation | Quantitative and quality targets | Reports submitted by health facility, then checked and verified by steering committee and performance purchasing agency (AAP). Patient surveys by community groups | Dutch Government and EU |
| Cambodia |  |  |  |  |  |  |  |  |  |  |
|  | Bhushan  2002 | Limited details of P4P; Contracting models were not performance-based | Randomly selected districts, each with population of 100,000-200,000 people. There were 3 contracted-in, 2 contracted-out, and four control districts. All districts at baseline had less than 20% of planned health activities as functional, and had extremely poor health service coverage. All districts were comparable with each other in terms of socioeconomic status and health service coverage | RCT | Contracting-out: contractor had full responsibility for delivery of specified services, employed staff, and had full management control.  Contracting-in: contractors provided management support to civil service health staff, operating costs through normal government channels.  Control areas - comparable operating budget supplement also made available. |  | FP knowledge, and FP prevalence rate |  |  |  |
|  | Soeters  2003 | Limited details of P4P | Pereang district: 176,000 inhabitants, 1x 60-bed referral hospital, 1x 40-bed district hospital, 15 health centres. Poor health status in Cambodia. Low outpatient attendance, health facilities lack adequate infrastructure, trained staff and sufficient funds to operate. Out of pocket expenditure 82% of total health care expenditure. Irregularly and poorly paid health staff, so many supplementing incomes informally. Health reform plan launched by Government in 1995 (health coverage plan, financing charter 1997, contractual pilot 1998). | Before and after study | Pereang district studied in this report followed the contracting-in model (contractors provided management support to civil service health staff, operating costs through normal government channels).  Contracts were also signed with each health worker for a basic monthly incentive payment 55%: punctuality incentive 15%, performance bonus 30%. | In the contracting-in model there was private management of public facilities, however there remained remote higher public management which meant that private managers did not have control of staff employment contracts etc. | Depo-provera and oral contraceptives | Performance bonus depended on the degree to which the monthly financial targets for the department or health facility were fulfilled. Non-financial targets were also specified, e.g. EPI coverage, no of TB inpatients, % of correct diagnosis and treatment (however no monitoring system, so unclear if used) | Unclear - monitoring and evaluation system put in place in 2001 | Asian Development Bank |
| Egypt |  |  |  |  |  |  |  |  |  |  |
|  | Huntington  2010 | No baseline data (no randomisation) | Those eligible for Basic benefit Package which included reproductive health services  Intervention: 4 PHUs  Control: 4 PHUs  Intervention facilities were better finished than controls but all were rated equal by quality accreditation scheme. District recognised as being most engaged with PBF was chosen as intervention. Some significant differences between patient populations of intervention and control groups in terms of baseline reproductive and wealth characteristics. Also large hospital near 1 intervention facility which charged less for reproductive health visits, so clinic saw less reproductive health consultations compared to control. | Controlled post-intervention study | Salary supplements in the form of incentive payments.  Controls received salary top-ups that were not incentive based. | Egyptian Health Sector Reform Programme also included a Family Health Fund (social security scheme). Investment in structures, equipment, training and quality assurance. |  | Quantity and quality targets |  |  |
| Haiti |  |  |  |  |  |  |  |  |  |  |
|  | Eichler  2006 | Insufficient detail and no FP results; Incentives not explicitly linked to changing patterns of performance | The programme covered a population of more than 3 million people.  The programme contracts 32 NGOs. | Before and after study (almost ITS) | NGOs paid partially on achieving defined performance targets related to attainment of health output targets and strengthening institutional capacity. NGOs at risk for 10.5% of the maximum potential payment they receive. |  | FP services | Specific targets not reported | Independent firm in 1st year, then self-reported by NGOs with random audits by external contractor | USAID |
|  | Eichler  (Ch.9, CGD) | No control, and not enough ITS points | Previous performance of NGOs was very heterogeneous.  Pilot was 3 NGOs that were chosen because they were considered ready to graduate into the PBF scheme.  Haiti – 80% of rural population lives on less than $1 per day. 40% of population has no access to basic health care services. | Before and after study | 3-phase project - pilot was change in payment based partly on performance. Next phases included additional NGOs and experimented with changes in design and implementation |  | Provision of modern FP methods at health facilities, and discontinuation rate for injectable and oral contraceptives. | 7 performance indicators (2 were FP: availability of modern methods and reduction in the rate of discontinuation) | Independent research firm evaluated pilot | USAID |
| India |  |  |  |  |  |  |  |  |  |  |
|  | Lim  2010  MoH  2006 | Before and after design. Not enough data points for ITS  No FP aspects reported | Total of 720,320 households from 611 districts, across different states in India (end survey) | Before and after study | JSY – Janani Suraksha Yojana Conditional cash transfers to incentivise women of low socioeconomic status, or from low performing states, to give birth in a health facility.  P4P to Accredited Social Health Activists (ASHAs) for institutional deliveries. ASHAs also instructed to facilitate at least 3 antenatal visits, neonatal immunisations, postnatal checks, and breastfeeding counselling, and promote FP (MoH) | Large demand side to programme | FP promotion (MoH) | Two payments made to ASHA:  The first at arrival at the health centre with the expectant mother, and the second after the ASHA has made the postnatal visit and the child has received BCG immunisation. (MoH)  FP incentives unclear | Submission of antenatal protocol and escorting woman to health facility triggers payment.  Monthly meetings of ASHAs (MoH) | Government of India |
| Liberia |  |  |  |  |  |  |  |  |  |  |
|  | Morgan  2012 | Not enough data points for ITS | NGOs supported government health facilities in 7 out of 15 counties (over 100 facilities). | Before and after evaluation | NGOs contracted to deliver services in public facilities and to help build the capacity of County Health Teams. Achievement of targets on 12 service delivery indicators were linked to a potential 6% bonus, while poor performance on 5 administrative and management indicators were linked to potential quarterly penalties. The bonus was distributed by the NGO to facility staff, NGO field staff, and County Health Teams and in some cases Community Health Workers. |  | All FP, particularly IUD | FP indicator rewarded was increases in Couple Years of Protection aggregate score for the entire country. 12 service delivery indicators and 5 administrative and management indicators. Quality tool for FP contains 15 standards. | Initially managed by USAID funded RBHS project. Now management being turned over to MOHSW with technical support from RBHS. Tools developed with assistance from CHAI and Jhpiego | USAID |
| Rwanda |  |  |  |  |  |  |  |  |  |  |
|  | Basinga  2011 | No FP outcome data reported | 55% of intervention households had health insurance. 52% of control households had health insurance (p0.02). 93% of intervention households were landowners, whereas 88% were in control. (not significant)  Context: Government immunisation campaign, larger health sector reforms including programmes to improve maternal and child health | Was designed to be RCT, but had to reassign districts following administrative boundary change | Payments made to facilities and used at each facility's discretion. P4P payments given for 14 key maternal and child health indicators |  | New contraceptive users, and number of women who received 1m resupply of contraceptives | 14 key maternal and child health output indicators | Monthly activity reports submitted by facilities, and quarterly requests for payment to District Steering Committee, who were responsible for verification of data and authorisation of payment. Unannounced audit visits to facilities every 3m. One-off tracking survey conducted by MoH in 2008. | World Bank |
| Sierra Leone |  |  |  |  |  |  |  |  |  |  |
|  | CORDAID  2014 | No data from before launch of P4P in April 2011 - evaluation found that data recording of very poor quality | Poor utilisation of health services, particularly by the poorest. Poorly resourced health facilities. 17% of the demand for FP was achieved in 2010.  Context: National Free Health Care Initiative (maternal and child care) | Prospective ITS | Payments made to facilities by limited indicators to improve quality of services being provided under the Free Health care Initiative | (problems with timely payments of incentives) | Modern FP | 6 output indicators, corrected for payment for crosscutting issues and remoteness of facilities. Quality component. | Facility reports, District Health management Teams and Council officials provides quarterly internal verification. CORDAID conducted external verification (evaluation of project). | World Bank |
